# Trait anxiety selectively amplifies reward learning under uncertainty, while N3 sleep attenuates it

**DOI:** 10.1016/j.isci.2026.117745

**Published:** 2026-10-01

**Authors:** Rakshita Deshmukh, Arjun Ramakrishnan

**Affiliations:** 1 Department of Biological Sciences and Bioengineering, Indian Institute of Technology Kanpur, Kanpur, Uttar Pradesh 208016, India; 2 Mehta Family Centre for Engineering in Medicine, Indian Institute of Technology Kanpur, Kanpur, Uttar Pradesh 208016, India; 3 Department of Cognitive Science, Indian Institute of Technology Kanpur, Kanpur, Uttar Pradesh 208016, India

**Keywords:** anxiety, learning under uncertainty, sleep-dependent learning, decision-making, individual differences, computational modeling

## Abstract

Adaptive learning under uncertainty requires distinguishing random variability (stochasticity) from true environmental change (volatility), yet how trait anxiety and sleep together influence it remains unclear. Across two preregistered experiments, participants completed a probabilistic reversal-learning task that independently manipulated volatility and stochasticity. Hierarchical Bayesian modeling showed that in stable but noisy environments, individuals with higher trait anxiety appeared to treat stochastic fluctuations as indicators of genuine change, resulting in over updating and lower evidence accumulation, while preserving normative trends in volatile environments. In a second experiment combining task performance with overnight sleep electroencephalography, greater N3 sleep was associated with reduced updating and higher evidence accumulation in stable environments among individuals with higher trait anxiety. These findings suggest that trait anxiety selectively alters how uncertainty is computationally represented, while N3 sleep appears to attenuate it. Together, these findings provide a computational framework linking anxiety, sleep, and adaptive learning under uncertainty.

## Highlights

• Trait anxiety selectively shapes reward learning under stochastic uncertainty
• Higher trait anxiety is linked to greater updating in stable, noisy environments
• Greater N3 sleep shifts reward learning toward task optimal values
• Greater N3 sleep increases evidence accumulation toward the best option

## Graphical abstract

**Figure:**
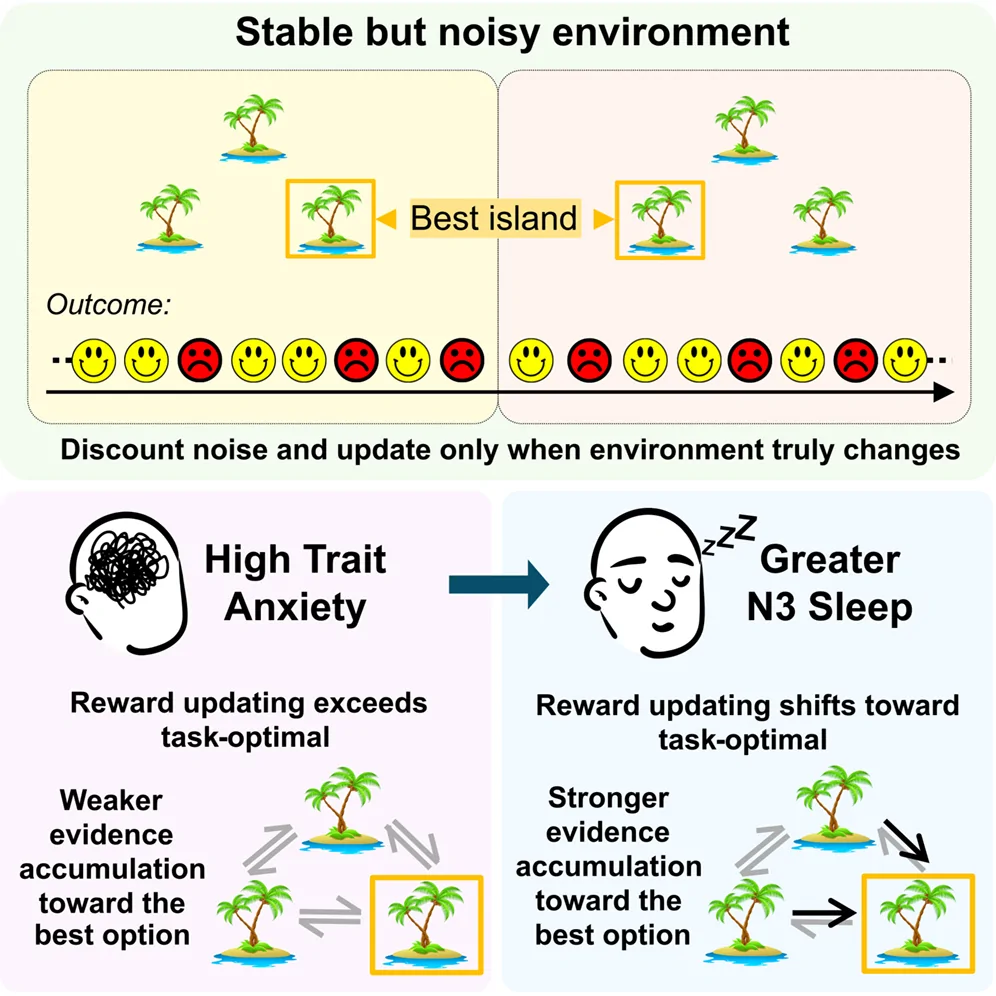

## Teaser

Biological sciences; Cognitive neuroscience; Natural sciences; Neuroscience

## Introduction

Adaptive behavior under uncertainty requires distinguishing stochasticity, random outcome variability under stable contingencies, from volatility, which reflects genuine environmental change. Normative learning models predict that learning rates should increase under volatility but decrease under high stochasticity to avoid overfitting noise.^1^^,^^2^ However, learning under uncertainty is also shaped by internal states that influence belief updating and learning-rate regulation, with anxiety representing a prominent and well-studied case.^3^^,^^4^^,^^5^ Anxiety is closely linked to anticipation of uncertain future events, with altered responses to uncertainty often conceptualized as intolerance of uncertainty (IU).^4^^,^^5^^,^^6^ Consistent with this view, contemporary theories suggest that anxious individuals show heightened sensitivity to ambiguous information and may assign greater significance to uncertain cues when inferring environmental states.^7^

Trait anxiety, a stable vulnerability factor for anxiety disorders, is consistently linked to differences in learning and decision-making under uncertainty. One influential view proposes that anxiety reflects altered processing of uncertainty, leading individuals to update beliefs differently when environmental outcomes are unpredictable. Prior work has associated these differences with heightened learning from negative outcomes, reduced evidence accumulation, and altered medial prefrontal and anterior cingulate regulation, collectively contributing to elevated estimates of environmental volatility.^5^^,^^7^^,^^8^^,^^9^^,^^10^^,^^11^^,^^12^ Anxiety has also been linked to differences in salience attribution and learning across reward and punishment domains.^13^^,^^14^ Critically, it remains unclear whether anxiety-related differences in learning under uncertainty reflect a global tendency to overestimate environmental change, or a more selective tendency to treat stochastic outcome variability as evidence for volatility while preserving sensitivity to true environmental change. Computational accounts propose that such differences may arise from altered subjective estimates of distinct forms of uncertainty, rather than from generalized learning deficits, highlighting the importance of dissociating stochasticity from volatility when characterizing anxiety-related differences in learning.^15^ A key unresolved question is whether anxiety-related differences reflect a generalized tendency to overestimate environmental uncertainty or more specific alterations in how particular forms of uncertainty are inferred and used to guide learning. Trait anxiety provides a natural test case for dissociating these possibilities.

Recent evidence suggests that some learners conflate stochasticity with volatility, increasing learning rates in noisy yet stable environments.^16^ Consistent with this idea, a study using a probabilistic reversal-learning task that independently manipulated stochasticity (outcome noise) and volatility (reversal frequency) found that individuals with higher trait anxiety exhibited learning patterns suggesting that increases in outcome noise were interpreted as increases in environmental volatility.^17^ These findings raise the possibility that anxiety-related differences arise from altered inference about specific forms of uncertainty rather than from a generalized learning deficit. However, previous anxiety research has primarily focused on volatility, with comparatively limited examination of how anxiety influences learning across different levels of stochasticity.

Learning-rate regulation and evidence accumulation are strongly state dependent, raising the possibility that internal physiological states such as sleep may modulate how uncertainty is inferred. Anxiety and sleep are tightly intertwined: sleep disruption exacerbates anxiety, while anxiety fragments sleep, jointly worsening outcomes,^18^^,^^19^ making sleep a compelling candidate for modulating anxiety-related differences in uncertainty processing. Sleep loss increases state anxiety and impairs prefrontal control, whereas non-rapid eye movement stage 3 (N3) sleep reduces morning state anxiety and supports synaptic plasticity and learning.^20^^,^^21^^,^^22^^,^^23^ Whether N3 sleep can specifically recalibrate learning-rate adjustment under uncertainty, rather than exerting global effects on affect or performance, remains unknown.

Across two preregistered experiments, we examined how trait anxiety shapes learning-rate regulation when stochasticity and volatility are independently manipulated in a three-option probabilistic reversal-learning task, and whether N3 sleep modulates these effects. Volatility was defined as the rate at which the underlying reward contingencies changed over time and was manipulated through reversal frequency. Stochasticity was defined as outcome uncertainty conditional on the current latent reward structure and was manipulated through the reward probability distributions assigned to the three options. This design allowed outcome noise to vary independently of environmental change. Although manipulated independently at the task-generation level, both uncertainty sources can influence belief updating and therefore produce partially overlapping behavioral signatures.

In experiment 1, we examined the effects of trait anxiety by testing two competing accounts. First, trait anxiety may be associated with globally elevated learning rates across uncertain environments, indicating greater updating to both stochastic variability and genuine contingency shifts. Under this account, learning rates would increase as environments become more stochastic and more volatile, irrespective of uncertainty type (H1a). Alternatively, trait anxiety-related differences may emerge specifically in noisy but stable contexts, reflecting a tendency to update excessively in response to stochastic outcome variability while preserving sensitivity to true volatility. This account predicts a context-specific elevation of learning rates when stochasticity is high, but volatility is low (H1b). We additionally preregistered that punishment learning rates would increase with uncertainty, particularly in individuals with higher trait anxiety, consistent with heightened sensitivity to negative feedback under uncertainty (H1c).

In experiment 2, we examined whether overnight N3 sleep modulates these computational phenotypes. If sleep has global effects on learning, anxiety-related differences should broadly diminish across conditions (H2a). Alternatively, if N3 sleep selectively attenuates uncertainty inference, effects should be strongest in contexts requiring discrimination between stochasticity and volatility (H2b). We further preregistered that N3 sleep would be associated with reductions in state anxiety and modulation of learning-rate parameters across sessions (H2c-d). To adjudicate between these possibilities, we combined hierarchical reinforcement-learning and drift-diffusion models to separately quantify learning-rate adjustment and evidence accumulation.

Together, these experiments tested whether trait anxiety is associated with global volatility hypersensitivity, selective misestimation of stochasticity as volatility, or valence-specific differences in punishment learning under uncertainty, and whether N3 sleep serves as a state-dependent mechanism capable of recalibrating these processes.

## Results

Across two preregistered experiments (AsPredicted, see additional resources) participants performed a three-option probabilistic reversal-learning task in which environmental uncertainty was independently manipulated along two dimensions: stochasticity (reward probability) and volatility (rate of contingency change), allowing these factors to be dissociated within a single task. In experiment 1, a 3 × 2 within-subject design varied stochasticity (low, medium, and high) and volatility (slow and fast). Fifty participants completed the task (*N* = 50, 20 women, 30 men, 22.32 ± 2.85 years), and trait anxiety was assessed using the STAI-Y (Figure 1A). In experiment 2, we examined sleep-dependent modulation of learning using a 2 × 2 × 2 within-subject sleep design, with task performance before and after sleep under low versus high stochasticity and slow versus fast volatility. Forty participants completed the task (Figure 1B, *N* = 40, 17 women, 23 men, 22.75 ± 3.04 years). Anxiety was assessed pre- and post-sleep and overnight sleep was monitored using wearable EEG (see STAR Methods).

**Figure 1:**
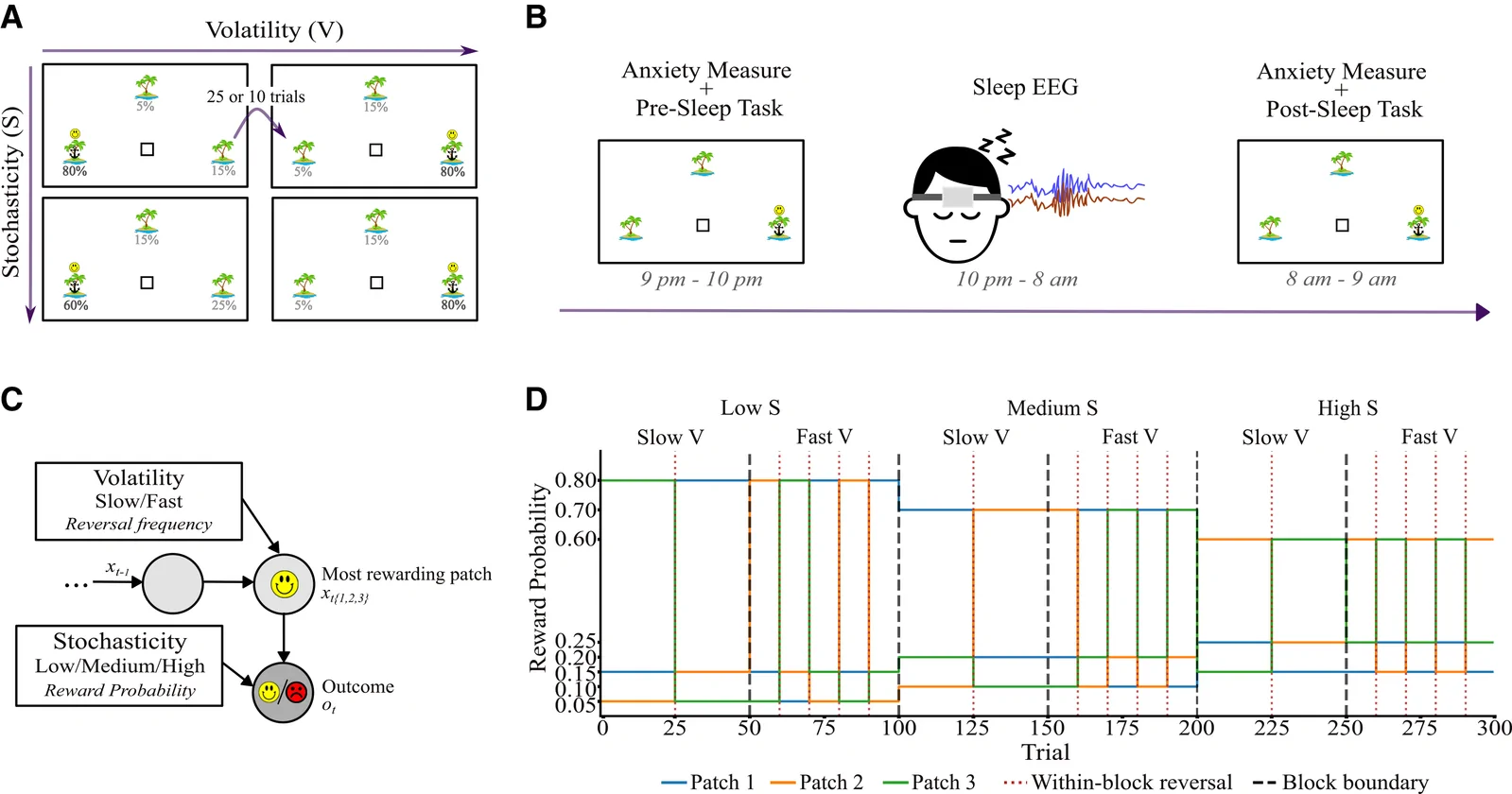
Experimental paradigm and manipulation of stochasticity and volatility (A) Experimental design: a three-option probabilistic reversal-learning task with simultaneous stochasticity (low, medium, high with most-rewarding patch reward probability: 80%, 70%, and 60%) and volatility (slow or fast, contingency changes every 25 or 10 trials in a 50-trial block, block order counterbalanced). On each trial, participants selected a patch from a central start position and received reward (smiley) or no-reward (sad) feedback to maximize earnings. Sample reward probabilities displayed on each patch, these probabilities were not shown to the participants. (B) Experiment 2 design: participants completed a three-option probabilistic reversal-learning task, similar to experiment 1, twice: pre- and post-sleep, with block order counterbalanced across sessions and participants. Task conditions manipulated stochasticity (low, high) and volatility (slow, fast), while anxiety was assessed using the STAI-Y scale before and after sleep. Participants slept in the laboratory, with sleep recorded using the Hypnodyne Zmax wearable EEG device. (C) Structure of task uncertainty. Stochasticity operated at the outcome level, determining the probability of receiving rewarded (smiley) or unrewarded (sad) outcomes from a selected option. Volatility operated at the environmental level, determining how frequently the identity of the most rewarding option (out of the 3 patches) changed across trials. Consequently, stochasticity affected the informativeness of outcomes, whereas volatility affected the stability of the reward structure. (D) Example reward probability schedules across task environments. Reward probability schedules for the three options across the six volatility × stochasticity environments. Stochasticity determined the reward probability distribution (low: 80–15–5, medium: 70–20–10, high: 60–25–15), whereas volatility determined reversal frequency (slow: every 25 trials; fast: every 10 trials). Colored solid lines indicate the reward probabilities of each option, red dotted lines indicate within-block reversals, and black dashed lines indicate block boundaries. Across all environments, reversals changed which of the three patches was currently the most rewarding while preserving the reward probability distribution specified by the stochasticity condition. *Note*: sample block order shown, across all participants block order was counterbalanced.

Specifically, stochasticity increased as the probability gap between the most-rewarding and lesser-rewarding options decreased (low: 80–15–5, medium: 70–20–10, and high: 60–25–15), making individual outcomes progressively less informative about the currently optimal option. Volatility was manipulated by varying the frequency of contingency reversals, with reversals occurring every 25 trials in slow-volatility conditions and every 10 trials in fast-volatility conditions within a 50-trial block. The factorial combination of reward probability distributions and reversal schedules ensured that increases in stochasticity reduced the informativeness of outcomes, whereas increases in volatility increased the rate of environmental change (Figures 1C and 1D). To verify that these task manipulations produced the expected computational demands, we simulated reinforcement-learning agents across a range of learning rates in each task environment and identified the reward-maximizing learning rate (see STAR Methods). As reported in subsequent sections, the resulting optimal learning rates increased with volatility and decreased with stochasticity, consistent with normative predictions regarding adaptive learning under uncertainty.

### High trait anxiety lowers reward accumulation in stochastic environments

We tested whether increasing environmental uncertainty reduced reward accumulation, and whether this effect was exacerbated in individuals with high trait anxiety (HTA; STAI-T ≥ 45) relative to low trait anxiety (LTA). Reward accumulation decreased with stochasticity (non-parametric ANOVA; *F*_2,276_ = 74.16, *p* < 0 .001, η^2^_*p*_ = 0.35) and volatility (*F*_1,276_ = 7.69, *p* < 0 .05, η^2^_*p*_ = 0.03), with graded effect of stochasticity (low > medium > high; all *p* < 0 .05; Table S1) and volatility levels (*p* < 0 .05).

Trait anxiety further modulated reward accumulation with a significant main effect (*F*_1,276_ = 4.08, *p* < 0 .05, η^2^_*p*_ = 0.02) and trait anxiety × volatility interaction (*F*_2,276_ = 4.87, *p* < 0 .05, η^2^_*p*_ = 0.02). This interaction was driven by reward accumulation by HTA individuals under slow volatility (simple effects; slow: *F*_1,142_ = 5.44, *p* < 0 .05,η^2^_*p*_ = 0.04), with no anxiety-related differences under fast volatility (fast: *F*_1,142_ = 0.01, *p* = 0.9,η^2^_*p*_ = 0.00). Consistent with this pattern, HTA participants accumulated fewer rewards than LTA under slow volatility (pairwise *t* tests:*t*_128_ = −2.3, 95% CI [−62.04, −4.69], *p* < 0 .05, *d* = −0.39).

Environment-specific analyses revealed that this trait anxiety-related difference was selective to high stochasticity, slow-volatility conditions, where HTA participants earned significantly fewer rewards than LTA counterparts (*t*_37_ = −2.61, 95% CI [−6.32, −0.7], *p* < 0.05, *d* = −0.77), with no group differences in other environments (Table S2).

To examine learning dynamics underlying these effects, we modeled trial-by-trial identification of the most rewarding option using Bayesian beta regression, indexing the proportion of these choices over time. Learning accuracy decreased with increasing stochasticity and volatility (Figure 2A). Critically, high trait anxiety selectively affected learning under slow volatility, in medium and high stochasticity environments (Figure 2A, left), with no trait anxiety-related differences under fast volatility (Figure 2A, right). HTA was associated with reduced learning of the most-rewarding patch (β = −0.17, 95% CI [−0.32, −0.01]), with this pattern being most evident under medium (β = −0.12, 95% CI [−0.18, −0.05]) and high stochasticity (β = −0.19, 95% CI [−0.25, −0.13]; Table S3).

**Figure 2:**
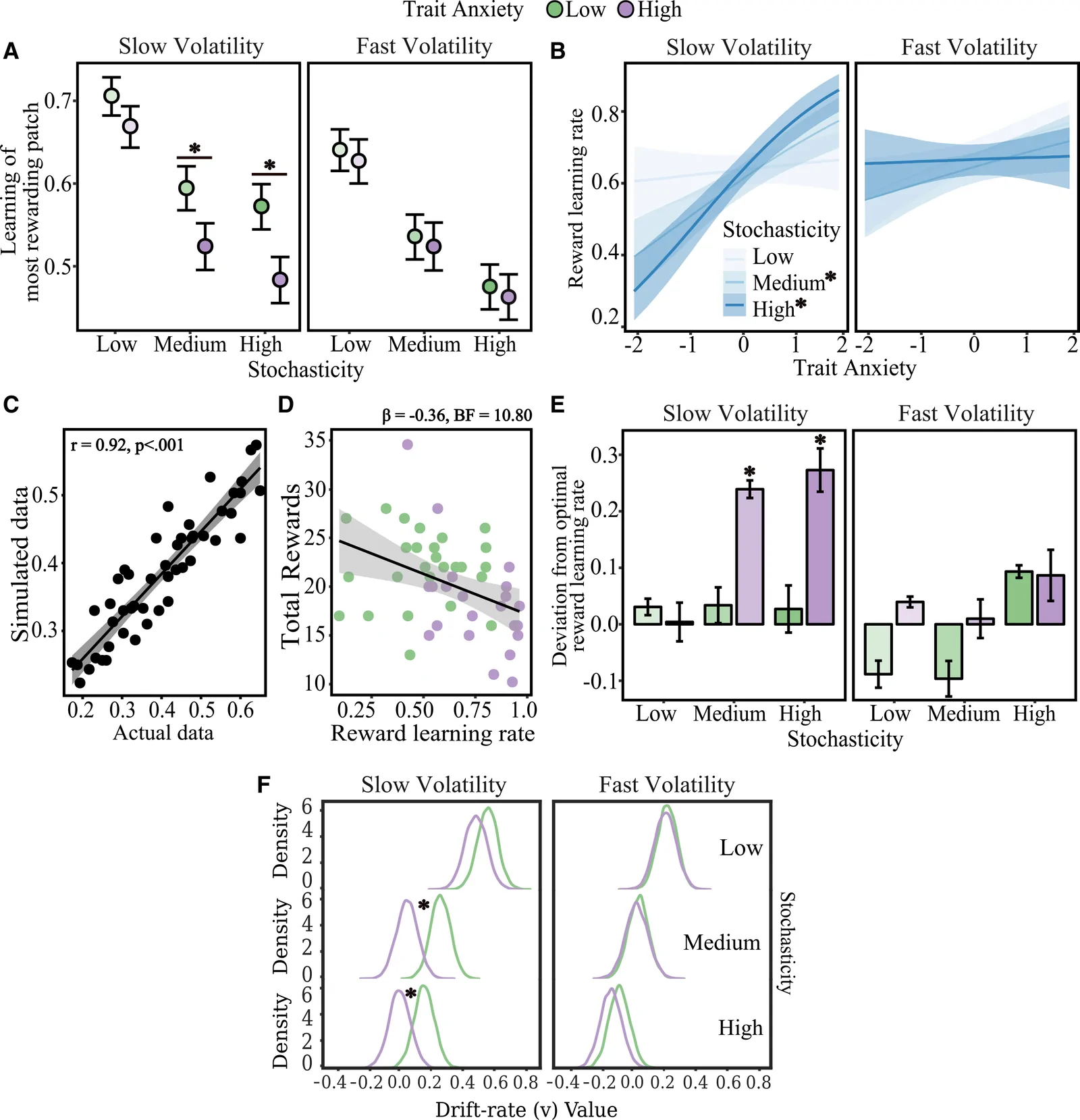
High trait anxiety is associated with reduced reward accumulation through elevated reward learning rates that exceed the task-optimal benchmark in stochastic environments (A) Posterior means (±SEM) of trial-wise learning of the most rewarding patch. As stochasticity and volatility increases, individuals with high trait anxiety (HTA) further lower learning in slow volatility but not fast volatility environments with this effect being largest in slow volatility-high stochasticity environments (Bayesian zero-one-inflated beta regression). (B) Posterior estimates of individual reward learning rates obtained from Hierarchical Bayesian reinforcement-learning modeling show that, under slow volatility, higher trait anxiety is associated with increasing reward learning rates as stochasticity increases, with anxiety modeled as a continuous variable (Bayesian beta regression). See also Figure S1. (C) Parameter recovery: Average participant proportion of patch switches in simulated and actual data for hierarchical Bayesian reinforcement learning model showing significant positive correlation (*p* < 0.05, Pearson’s correlation), suggesting that the model captured the data well. (D) Participant environment-specific total rewards show higher reward learning rates are associated with significantly lower rewards in slow volatility-high stochasticity environment with HTA being clustered toward this regime (*BF* > 1, Bayesian correlation). See also Figures S3A–S3C. (E) Bar plots show the mean (±SEM) deviation from the optimal value (individual reward learning rate-optimal value) for each environment. HTA individuals significantly exceeded the task-optimal benchmark in slow-volatility, high-stochasticity environments (BF > 1; Bayesian one-sample *t* tests), but not in fast volatility-high stochasticity environments. See also Figure S2A and S2B. (F) Posterior distribution of drift-rates from hierarchical drift-diffusion modeling shows that while higher stochasticity and fast volatility lower drift-rates, HTA show even lower drift-rates in slow volatility-high stochasticity environments, with values centered around zero (*q* < 0.05, comparing overlap proportions) but there is no difference between the two trait anxiety groups in fast volatility environments. *Note*: asterisks (✽) denote significant effects based on inferences from Bayesian statistics. Data are shown for 48 participants; two participants were excluded from this analysis per exclusion criteria (see STAR Methods). For all regression models, reference levels were set as low stochasticity, slow volatility, and LTA.

Together, these findings indicate that high trait anxiety selectively lowers performance in stable environments characterized by high stochasticity.

### High trait anxiety is associated with elevated reward learning rates under stochastic environments

We preregistered the hypothesis (H1a, H1c) that higher trait anxiety (HTA) would be associated with elevated punishment relative to reward learning rates under conditions of high uncertainty. Given the observed association between HTA and lower performance in slow volatility environments, we first examined whether variation in punishment learning contributed to this learning behavior in stable environments among HTA (H1b). Contrary to this prediction, HTA was associated with higher reward learning rates under slow volatility, selectively in medium and high stochasticity environments.

To estimate learning rates, we fit a 5-parameter hierarchical Bayesian reinforcement-learning model with separate learning rates for reward and punishment (see STAR Methods). The model provided a good account of behavior (Figure 2C). Under slow volatility, HTA individuals exhibited elevated reward learning rates in medium (95% HDI [0.11 and 0.51]) and high-stochasticity conditions (95% HDI [0.03 and 0.39]), Tables S4 and S5). This effect was replicated at the individual-level using a Bayesian beta regression (medium: β = −0.41, 95% CI [−0.73, −0.10]; high: β = −0.83, 95% CI [−1.16, −0.5], Tables S6 and S7), and was robust when trait anxiety was modeled as continuous (Figure 2B left, increasing from left to right) or categorical variable (Figure S1). Notably, this finding runs counter to normative expectations, which predict lower learning rates in stable environments as stochasticity increases. However, under high volatility, reward learning rates did not vary as a function of trait anxiety (Figure 2B, right).

We next tested whether elevated learning rates were maladaptive in stable stochastic environments. Higher reward learnings rates were associated with lower reward accumulation in the slow-volatility, high-stochasticity condition (Bayesian correlation: β = −0.36, 95% CI [−0.57, −0.09], BF = 10.80) with HTA individuals tending to cluster in the region characterized by higher learning rates and lower reward accumulation (Figure 2D). Punishment learning rates were not associated with reward accumulation, indicating that elevated reward learning, rather than punishment learning, was associated with lower reward accumulation in this condition (Table S8).

To benchmark learning behavior against normative expectations, we simulated optimal learning rate values for each task environment (see STAR Methods). As expected, optimal reward learning rates increased with volatility and decreased with stochasticity (two-way ANOVA, stochasticity: *F*_2,294_ = 15.49, *p* < 0 .001, η^2^_*p*_ = 0.10; volatility: *F*_1,294_ = 41.18, *p* < 0 .001, η^2^_*p*_ = 0.12, Figure S2A). Although volatility and stochasticity were manipulated independently at the task-design level, we observed a small volatility × stochasticity interaction in the simulations (*F*_2,294_ = 3.51, *p* < 0.05, η^2^_*p*_ = 0.02). Follow-up analyses showed that the overall pattern was preserved across task environments, with optimal learning rates generally decreasing with increasing stochasticity and increasing under fast relative to slow volatility (Figure S2B). Relative to these benchmarks, HTA participants exhibited higher learning rates than the optimal benchmark in slow volatility, medium stochasticity (Bayesian one-sample *t* tests: β = 0.24, 95% CI [0.20, 0.27], BF = 1.04e+10) and high stochasticity environments (β = 0.26, 95% CI [0.18, 0.35], BF = 33200, Figure 2E, left), but not in fast volatility environments (Figure 2E, right, medium: β = 0.01, 95% CI [−0.05, 0.07], BF = 0.23; high: β = 0.08, 95% CI [−0.02, 0.17], BF = 1.04).

Punishment-related parameters did not account for anxiety-related differences in performance. Punishment learning rates remained below task-optimal levels in both HTA and LTA participants and did not differ substantially between groups under slow volatility, indicating that punishment learning did not contribute to reward maximization in this task (Figures S3A–S3C; Table S11). Similarly, punishment sensitivity was below task-optimal levels in both groups. Although both groups differed from task-optimal values across other parameters, HTA participants exhibited greater reward sensitivity, higher randomness in responding (noise), and elevated reward learning rates than LTA participants in the slow-volatility, high-stochasticity environment (Figure S3C). Together, these findings suggest that HTA individuals adopted a more reward-focused strategy, whereas group differences in punishment-related parameters did not explain performance differences.

Finally, we tested whether these learning differences reflected altered valuation of the most rewarding option. Using a hierarchical drift-diffusion model, we found that under slow volatility and high stochasticity, HTA participants exhibited reduced drift rates, centered near zero relative to LTA participants (Figure 2F, left, hierarchical drift-diffusion model, high: 95.24%, *q* < 0.05, medium: 98.97%, *q* <0.05; Tables S9 and S10), along with higher decision boundaries in this environment (95.08%, *q* < 0.05). Bias and non-decision time did not differ between groups.

This trait anxiety-related difference was selective to slow-volatility environments and was absent under fast volatility, where neither learning rates nor evidence accumulation differed between anxiety groups (Figure 2F, right).

Collectively, these findings indicate that high trait anxiety is associated with elevated reward learning rates in stable yet noisy environments, resulting in learning rates that exceed the task-optimal benchmark and suggesting that stochastic outcome variability may be treated as evidence of environmental change.

### N3 sleep is associated with lower morning state anxiety and better post-sleep performance in high trait anxious individuals

In experiment 1, we confirmed a bidirectional relationship between trait anxiety and sleep quality: higher trait anxiety was associated with poorer subjective sleep quality (Sleep Condition Indicator; Spearman’s r(46) = −0.41, *p* < 0.05), consistent with prior reports linking anxiety to sleep.^19^

For Experiment 2, we preregistered the hypotheses (H2c) that greater N3 sleep would predict lower next-day state anxiety based on previous findings,^20^ and that LTA participants would show greater N3 sleep and larger overnight state anxiety reduction than HTA individuals. Across participants, greater N3 sleep was associated with larger evening-to-morning reduction in state anxiety, with no comparable associations for N1, N2, or REM sleep. (Pearson’s correlation, r(38) = 0.36, *p* < 0.05, Table S12). Linear regression revealed significant main effect of N3 sleep (β = 0.46, 95% CI [0.15, 0.77], t_32_ = 3.01, *p* < 0.05) and an interaction with trait anxiety (β = 0.43, 95% CI [0.06, 0.79], t_32_ = 2.39, *p* < 0.05, Table S13).

Contrary to our hypothesis, HTA participants showed the greatest anxiolytic benefit from N3 sleep (Figure 3A, in purple, increasing from left to right).

**Figure 3:**
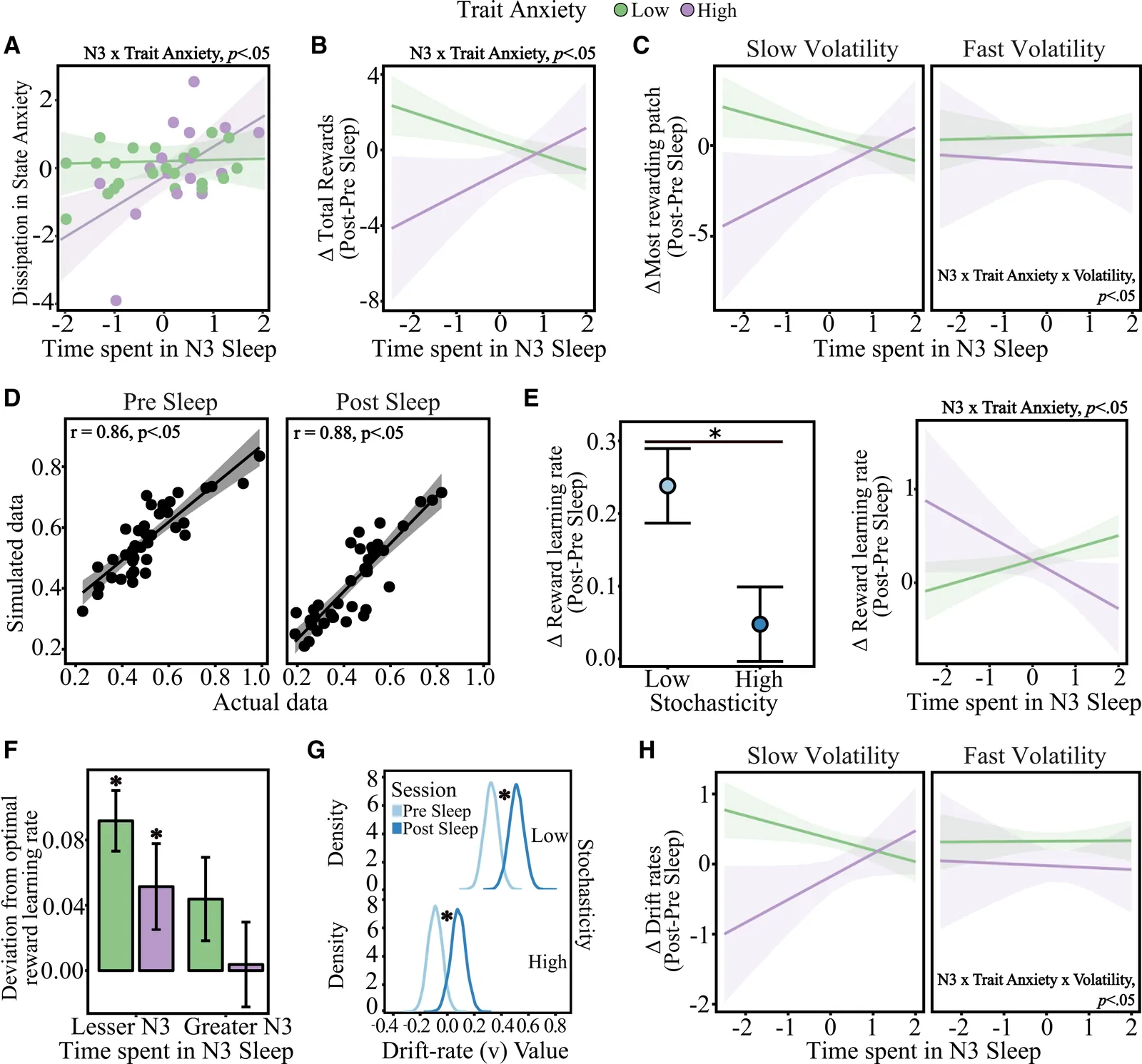
N3 sleep is associated with improved post-sleep performance in high trait anxiety (HTA) by reducing reward learning rates and improving patch valuation under slow volatility environments (A) N3 sleep significantly interacts with trait anxiety in the dissipation of state anxiety (pre-post sleep state anxiety score), with higher values indicating positive post-sleep reductions in state anxiety; greater N3 sleep is associated with increasingly positive dissipation of morning state anxiety in HTA (*p* < 0.05, linear regression). (B and C) The benefit of N3 sleep for HTA extends to task performance, with greater N3 sleep associated with higher post-sleep rewards overall and improved identification of the most rewarding patch in slow-volatility environments (*p* < 0.05, linear mixed-models). (D) Parameter recovery: average participant proportion of patch switches in simulated and actual data per task session for hierarchical Bayesian reinforcement learning model showing significant positive correlation (*p* < 0.05, Pearson’s correlation), suggesting that the model captured the data well. (E) Individual level reward learning rates from the hierarchical Bayesian reinforcement learning model shows that post-sleep reward learning rate decreases in high stochasticity (left), with greater N3 sleep reducing HTA’s overall reward learning rates (right) (*p* < 0.05, linear mixed-models). (F) Bar plots show the mean (±SEM) deviation from the optimal value (individual reward learning rate-optimal value) for post-sleep session. Greater N3 sleep was associated with reward learning rates that more closely matched the task-optimal benchmark (*BF* < 1, Bayesian one-sample *t* tests). In contrast, lower N3 sleep (categorical mean split) was associated with learning rates that exceeded the task-optimal benchmark in both trait anxiety groups, suggesting that N3 sleep may influence post-sleep reward learning, particularly in HTA (*BF* > 1, Bayesian one-sample *t* tests). (G) Posterior distribution of drift-rates from hierarchical drift-diffusion modeling shows post-sleep drift rates increase across environments (*q* < 0.05, comparing posterior proportions). (H) At the individual level, greater N3 sleep increases higher post-sleep drift rates in HTA, particularly in slow-volatility environments (*p* <0.05, linear mixed-models). Fast volatility environments remain unaffected by anxiety or sleep. *Note*: asterisks (✱) denote significant effects based on frequentist statistics, whereas ✽ indicates inferences from Bayesian statistics. Variables were normalized; high and low trait anxiety reflect a mean split for plotting, with continuous values used for statistical analyses. Data are shown for 40 participants. For all regression models, reference levels were set as low stochasticity and slow volatility.

We next examined whether sleep-related anxiety reduction translated into improved task performance (H2a-b). We preregistered that poor sleep would lower performance, particularly in HTA individuals. Across participants, post-sleep reward accumulation was higher than pre-sleep (ANOVA: *F*_1,312_ = 8.907, *p* < 0.05,η^2^_*p*_ = 0.03; Post-hoc *t* test:t_159_ = 3.33, *p* < 0.05, *d* = 0.26, 95% CI [0.68, 2.66]), while stochasticity and volatility reduced rewards across both sessions (Table S14). Critically, greater N3 sleep predicted higher post-sleep reward accumulation in HTA participants (Figure 3B, linear mixed-model, β = 0.46, 95% CI [0.03, 0.88], t_133_ = 2.13, *p* < 0.05; Tables S15 and S16). This benefit was driven by improved ability to identify the most rewarding patch under slow volatility (Figure 3C left, in purple, increasing from left to right**;** linear mixed-model: β = −0.49, 95% CI [−0.92, −0.07], t_120_ = −2.28, *p* <0.05; Table S17), but not under fast volatility (Figure 3C, right).

Together, these results indicate that greater N3 sleep is associated with reduced morning state anxiety and enhanced performance among HTA individuals, particularly in stable but noisy environments.

### N3 sleep attenuates high trait anxious individual’s elevated reward learning rates

We preregistered that poor sleep would exacerbate punishment learning in high trait-anxious (HTA) individuals under high uncertainty, whereas good sleep would enhance reward learning in low trait-anxious (LTA) individuals (H2d). However, experiment 1 instead revealed that HTA individuals exhibited *elevated reward learning rates* in slow volatility, high stochasticity environments, and experiment 2 showed that N3 sleep reduced morning state anxiety and improved post-sleep performance in HTA participants. These findings motivated tests of whether N3 sleep selectively modulates reward learning in environments where HTA individuals exhibit elevated updating.

Reward learning rates were estimated using a hierarchical Bayesian reinforcement-learning model, which provided a good account of behavior pre- and post-sleep (Figure 3D). Across participants, reward learning rates decreased from pre-to post-sleep under high stochasticity, consistent with normative learning-rate adjustment (Figure 3E, left, linear mixed model: stochasticity: β = −0.92, 95%CI [−1.16, −0.69], *p* < 0.05). Importantly, greater N3 sleep was associated with reduced post-sleep reward learning rates in HTA individuals across all environments (Figure 3E, right, in purple, decreasing from left to right**;** N3 × trait anxiety: β = −0.45, 95% CI [−0.86, −0.03], *p* < 0.05; Tables S18–S20). Focusing specifically on slow volatility-high stochasticity environment where HTA showed elevated learning rates in experiment 1, greater N3 sleep significantly reduced post-sleep reward learning rates in HTA individuals (linear regression: β = −0.09, 95% CI [−0.17, −0.01], *p* < 0.05).

To assess whether N3 sleep shifted learning rates toward task-optimal values, we compared pre- and post-sleep learning rates with simulated optimal values before and after sleep. In the pre-sleep session both HTA and LTA groups that significantly differed from the optimal reward learning rate (Bayesian one-sample *t* tests: HTA: β = −0.07, 95% CI [−0.13, −0.02], BF = 5.15; LTA: β = −0.07, 95% CI [−0.14, −0.04], BF = 37.74). But, post-sleep, greater N3 sleep was associated with learning rates that were near optimal values for both groups, whereas less N3 sleep was associated with significant differences from optimal values (Figure 3F, Bayesian one-sample t-tests: HTA-more N3: β = 0.004, 95% CI [−0.06, 0.07], BF = 0.30; LTA-more N3: β = 0.04, 95% CI [−0.02, 0.10], BF = 0.62; HTA-less N3: β = 0.04, 95% CI [0.01, 0.08], BF = 3.22; LTA-less N3: β = 0.08, 95% CI [0.02, 0.14], BF = 11.14). Thus, greater N3 sleep shifted HTA’s elevated reward learning rates toward optimal values.

Finally, we tested whether N3 sleep improved reward learning in HTA individuals by enhancing valuation of the most rewarding patch. Post-sleep drift rates were significantly higher than pre-sleep across all environments (Figure 3G, hierarchical drift-diffusion model, low-slow = 99.18%, *q* < 0.05; low-fast = 99.93%, *q* < 0.05; high-slow = 98.63%, *q* < 0.05; high-Ffast = 97.28%, *q* < 0.05). Moreover, greater N3 sleep significantly interacted with trait anxiety overall and specifically under slow volatility environments in increasing post-sleep drift-rates in HTA individuals (Figure 3H, left, in purple, increasing from left to right; linear mixed model: N3 × trait anxiety: β = 0.48, 95% CI [0.03, 0.92], *p* < 0.05; N3 x trait anxiety × volatility: β = −0.51, 95% CI [−0.93, −0.08], *p* < 0.05; Tables S21 and S22). These changes suggest greater evidence accumulation in favor of the most rewarding patch by HTA individuals following sleep.

Together, these findings indicate that N3 sleep selectively influences reward learning in high trait anxiety, bringing learning rates closer to task-optimal values and increasing valuation of the most rewarding option during decision-making.

## Discussion

Although anxiety-related differences in learning under uncertainty are well documented,^24^^,^^25^ the computational mechanisms underlying these differences remain unclear. By jointly manipulating environmental stochasticity and volatility, we observed a selective pattern of performance differences in HTA individuals, consistent with an increased tendency to interpret stochastic reward fluctuations as indicators of actual environmental change. While HTA individuals remained sensitive to true environmental volatility,^26^ they appeared less likely to discount stochastic outcome variability appropriately in relatively stable environments. Consequently, they exhibited elevated reward learning rates and slower evidence accumulation in stable yet noisy environments, consistent with more frequent belief updating.^27^ Notably, these anxiety-related differences were selective to environments requiring discrimination between stochastic fluctuations and genuine environmental change. Importantly, greater N3 sleep was associated with an attenuation of these trait anxiety-related differences, bringing learning rates closer to task-optimal values and influencing post-sleep decision-making.

Computational modeling revealed that while high learning rates are adaptive in volatile environments, high trait anxious individuals maintained elevated reward learning rates even in stable but noisy conditions, resulting in greater updating under stochasticity. This pattern is consistent with prior work suggesting that high trait anxiety is associated with increased state inference,^28^ whereby surprising outcomes are more readily interpreted as signals of environmental change. More broadly, it aligns with evidence that stochastic fluctuations can be treated as informative rather than incidental,^16^ resulting in reduced discrimination between signal and noise^9^ and elevated estimates of environmental volatility in individuals with high trait anxiety.^29^^,^^30^ Recently, a study manipulating volatility and outcome noise similarly found that individuals with higher anxious traits increased learning rates under high noise when volatility was also high, suggesting a tendency to treat stochastic fluctuations as evidence of environmental change.^17^ However, whereas those effects were primarily observed under high-volatility conditions, the present findings emerged in stable but noisy environments, suggesting that high trait anxious individuals may treat stochastic fluctuations as signals of environmental change even when changes are slow.

Differences in learning rate were accompanied by differences in the decision process itself. Consistent with prior links between anxiety and differences in decision-making under cognitive demand,^11^^,^^31^^,^^32^^,^^33^ these effects were accompanied by lower drift rates, suggesting slower accumulation of reward evidence during decision-making.^34^ Notably, the observed differences were reward-driven rather than punishment-driven, challenging dominant accounts that attribute anxiety-related learning differences primarily to enhanced punishment sensitivity or threat learning. Even in the absence of explicit penalties, high trait anxious individuals appeared more likely to treat noisy rewards as informative about environmental change. This interpretation is consistent with evidence that anxiety amplifies sensitivity to salient monetary incentives under uncertainty,^4^^,^^13^^,^^35^ or whichever outcome appears most informative.^14^^,^^36^

Consistent with previous work, greater N3 sleep was associated with reductions in morning state anxiety,^20^ which held true in our data as well. Extending these findings, we observed that HTA individuals appeared to derive the greatest benefit from N3 sleep. This benefit also extended to improved post-sleep performance in HTA participants, yielding higher rewards and more accurate identification of the optimal option. While N3 sleep has been linked to adaptive learning across domains, including motor learning and cognitive flexibility,^23^^,^^37^^,^^38^ in our task greater N3 sleep was associated with selective changes in learning rates and evidence accumulation, bringing learning behavior in HTA individuals closer to patterns expected from the distinction between stochastic noise and environmental change. Whereas sleep deprivation studies have reported exaggerated reward and threat reactivity, reduced sensitivity to environmental volatility, and altered belief updating,^39^^,^^40^^,^^41^ our findings suggest that greater N3 sleep may influence how uncertainty is incorporated into learning and decision making particularly in individuals with high trait anxiety.

Overall, these findings suggest that high trait anxiety is associated with a tendency to treat stochastic fluctuations as indicative of environmental change, resulting in elevated reward learning rates and slower evidence accumulation in stable environments. In contrast, greater N3 sleep was associated with changes in these computational processes, bringing reward learning rates closer to task-optimal values and increasing evidence accumulation. Because stochasticity-volatility trade-offs are fundamental to many real-world learning problems, these findings may extend beyond the present task and highlight sleep as a potential modulator of the relationship between anxiety, learning, and decision-making under uncertainty.

### Limitations of the study

Several considerations help define the scope of these findings. Although we had no *a priori* reason to expect sex- or gender-related differences, the present study had limited statistical power to assess their effects on learning under uncertainty and sleep among individuals with high trait anxiety. Because N3 sleep was not experimentally manipulated, its restorative effects remain partially correlational, motivating future causal tests using targeted modulation of N3 sleep. In addition, IU, a construct closely related to trait anxiety and implicated in learning under uncertainty,^42^^,^^43^^,^^44^ was not assessed. Consequently, we cannot determine whether the observed effects are specific to trait anxiety or reflect variance shared with IU, highlighting the need for future studies to examine their unique and overlapping contributions. Thus, the present findings should be interpreted as reflecting trait anxiety-related variation, rather than effects uniquely attributable to trait anxiety. Furthermore, unmeasured circadian and chronotype factors may have influenced pre-versus post-sleep performance, highlighting the importance of jointly considering sleep and circadian contributions to learning under uncertainty. Notably, the observed effects were selective to environments requiring appropriate discounting of stochastic noise, arguing against nonspecific arousal or vigilance accounts.

## Resource availability

### Lead contact

Requests for further information and resources should be directed to and will be fulfilled by the lead contact, Arjun Ramakrishnan (arjunr@iitk.ac.in).

### Materials availability

This study did not generate new unique reagents.

### Data and code availability

• All de-identified human behavioral and summary statistics from sleep EEG data have been deposited at Zenodo: https://doi.org/10.5281/zenodo.18138007. They are publicly available as of the date of publication.
• All original code has been deposited at Zenodo: https://doi.org/10.5281/zenodo.18138007, and is publicly available as of the date of publication.
• Any additional information required to reanalyze the data reported in this paper is available from the lead contact upon request.

## Acknowledgments

We thank Chetan Kandpal for technical assistance in sleep data collection. This work was supported by the Science and Engineering Research Board start-up research grant SRG/2020/001723 (to A.R.), and DBT Wellcome Trust Intermediate Fellowship IA/I/20/2/505204 (to A.R.). We gratefully acknowledge the IISc-Pratiksha Trust’s Brain, Computer and Data Science Initiative for awarding a Senior Student Research Associateship to R.D.

## Author contributions

Conceptualization, R.D. and A.R.; methodology, R.D. and A.R.; investigation, R.D. and A.R.; data curation, R.D.; formal analysis, R.D.; writing – original draft, R.D.; writing – review and editing, R.D. and A.R.; funding acquisition, A.R.; resources, A.R.; supervision, A.R.

## Declaration of interests

The authors declare no competing interests.

## STAR★Methods

### Key resources table

**Table:** 

REAGENT or RESOURCE	SOURCE	IDENTIFIER
**Deposited data**		

Behavioural and physiological data; analysis code	This paper; Zenodo	https://doi.org/10.5281/zenodo.18138007

**Software and algorithms**		

R version 4.4.2	R Core Development Team	https://www.r-project.org/; RRID: SCR_001905
Python version 3.12.7	Python Software Foundation	https://www.python.org/ ; RRID: SCR_008394
hBayesDM	Ahn et al.,^45^	https://ccs-lab.github.io/hBayesDM/index.html
HDDM	Wiecki et al.,^46^	https://hddm.readthedocs.io/en/latest/intro.html
YASA (Yet Another Spindle Algorithm)	Vallat & Walker^39^	https://github.com/raphaelvallat/yasa

**Other**		

Hypnodyne ZMax	EEG Headband	https://hypnodynecorp.com/zmax.php

### Experimental model and study participant details

Both experiments were preregistered on AsPredicted (See additional resources). The study was approved by the Institute Ethics Committee at the Indian Institute of Technology Kanpur (IITK/IEC/2020-21/II/10), and all participants provided written informed consent prior to participation. Participants were recruited from the campus community of the Indian Institute of Technology Kanpur (100% Asian). Experiment 1 initially enrolled 50 participants (20 women, 30 men; age: 22.32 ± 2.85 years). Two participants were excluded due to excessive sleepiness (Stanford Sleepiness Scale [SSS] ≥ 3), resulting in a final sample of 48 participants (19 women, 29 men; age: 22.44 ± 2.83 years). Experiment 2 included 40 participants (17 women, 23 men; age: 22.75 ± 3.04 years), all of whom met the inclusion criteria. Participants received fixed monetary compensation in addition to performance-based bonuses. In Experiment 1, participants received INR 30 plus a performance-based bonus of up to INR 50. In Experiment 2, participants received INR 350 for overnight participation plus a performance-based bonus of up to INR 100.

### Method details

#### Sample size estimation

Sample size for Experiment 1 was determined through an *a priori* power analysis based on pilot data using the *simr* package in R,^47^ which indicated that 40–50 participants would be required to achieve 80% power. Accordingly, a target sample of 50 participants was selected to accommodate potential exclusions. For Experiment 2, sample size expectations were informed by prior sleep studies,^20^^,^^23^^,^^48^ which typically included 14–32 participants. An upper recruitment limit of 40 participants was set to allow for potential exclusions while ensuring an adequate final sample.

#### Exclusion criteria

Across both experiments, participants were excluded if they reported excessive sleepiness (SSS ≥ 3) or failed to sample all available choice options during the task. Additional exclusion criteria for Experiment 2 included age outside the range of 18–35 years; a history of sleep, neurological, or Axis I psychiatric disorders; substance abuse; closed head injury; or use of medications known to affect sleep or cognition. Participants were instructed to abstain from alcohol, recreational drugs, and caffeine on the day of testing in accordance with standard sleep study procedures.^20^

#### Questionnaires

Participants completed the Stanford Sleepiness Scale (SSS), State–Trait Anxiety Inventory (STAI-Y), and Sleep Condition Indicator (SCI) to assess alertness, anxiety, and subjective sleep quality, respectively. Questionnaires were administered following informed consent and prior to task commencement.

The SSS^45^ assessed momentary alertness and was used solely as a screening measure; participants reporting SSS scores ≥3 were excluded and these scores were not included in subsequent analyses. The STAI-Y assessed both state and trait anxiety.^46^ Trait anxiety was analyzed both continuously and categorically, with participants classified as high trait anxious (HTA; STAI-T ≥45) or low trait anxious (LTA), consistent with prior literature.^12^^,^^49^^,^^50^ K-means clustering independently identified two clusters with a boundary at STAI-T=45, supporting this threshold. In Experiment 1, state anxiety scores ranged from 20–60 (34 ± 9) and trait anxiety scores from 26–60 (44 ± 8.8). In Experiment 2, trait anxiety scores ranged from 30–72 (44.35 ± 10.01), while state anxiety scores ranged from 21–60 (33.45 ± 8.99) before sleep and 20–63 (32.43 ± 9.62) after sleep. Subjective sleep quality was assessed using the SCI^51^ (range: 5–32; mean = 22.98 ± 5.9 across both experiments).

In Experiment 2, participants additionally completed a post-sleep questionnaire^51^^,^^52^ assessing sleep experience, dream occurrence and valence, morning sleepiness, refreshment, and wearable EEG device comfort. Participants reported a mean sleep onset latency of 35.0 ± 24.88 min, 1.85 ± 1.23 nocturnal awakenings, and a perceived sleep duration of 7.02 ± 1.47 h. Mean ratings indicated good sleep quality (5.15 ± 1.05), low morning sleepiness (2.63 ± 1.53), high refreshment (5.25 ± 1.25), and good device comfort (5.23 ± 1.54). These post-sleep measures were collected for descriptive purposes and were not included in subsequent analyses.

#### Experimental design

Participants performed a three-option probabilistic reversal-learning task framed as a fishing game. On each trial, they selected one of three fishing islands (“patches”) to maximize rewards, with feedback presented visually as a smiley emoji for a successful catch and a sad emoji for no catch. Participants were informed that some fishing locations were generally more rewarding than others and that contingencies could change over time as fish moved between islands. However, they were not informed about the exact reward probabilities or reversal structure. This cover story and feedback structure were identical across all task conditions and experiments.

Within this framework, environmental uncertainty was independently manipulated along two dimensions: stochasticity (reward probability) and volatility (rate of contingency change). Each environment consisted of 50 trials. Stochasticity was manipulated by varying the reward probability of the optimal patch (80%, 70%, or 60%), with reciprocal probabilities assigned to the remaining options (15% and 5%, 20% and 10%, or 25% and 15%, respectively). Volatility was manipulated by reversing reward contingencies every 10 trials (fast) or 25 trials (slow). Reward probabilities were randomized across options, and participants completed five practice trials before beginning the experimental task.

In Experiment 1, a 3 × 2 within-subject design manipulated stochasticity (Low, Medium, High) and volatility (Slow, Fast). Following questionnaire completion, participants completed six 50-trial blocks (300 trials total), with block order counterbalanced and 30-s breaks between blocks (Figure 1A). In Experiment 2, a 2 × 2 × 2 within-subject sleep design manipulated stochasticity (Low, High) and volatility (Slow, Fast), with task performance assessed before and after sleep (Figure 1B). Participants completed four 50-trial blocks per session (400 trials total). The sleep laboratory was maintained at a constant temperature of 26°C.

#### Sleep EEG recording and preprocessing

Sleep was recorded overnight using the Hypnodyne ZMax, a wearable EEG system validated against standard polysomnography (PSG) for whole-night sleep monitoring.^53^^,^^54^ Following completion of the pre-sleep task, participants’ foreheads were cleaned, a new disposable electrode patch was applied, and the headband was secured centrally on the forehead using an adjustable strap. Wireless recording was initiated using ZMax’s HDRecorder software, and signal quality was verified through a brief calibration procedure involving head movements and eye blinks. Two frontal bipolar EEG channels (F7-FPz and F8-FPz) were recorded at 256 Hz, and sleep was recorded continuously overnight without data loss. To assess concordance between channels, preregistered sleep variables (total sleep time [TST], N3 duration, and sleep onset latency [SOL]) were compared between left and right electrodes using Wilcoxon rank-sum tests. No significant differences were observed (TST: W=814, *p*=.90; N3: W=848, *p*=.70; SOL: W=731.5, *p*=.50); therefore, all subsequent analyses were conducted using the left channel (F7-FPz; EEG-L).

Sleep staging was performed in 30-s epochs according to AASM criteria using YASA, an open-source Python tool validated against expert human scoring and demonstrating high generalizable accuracy without systematic bias across sleep stages.^55^ Raw EEG data were downsampled to 100 Hz and band-pass filtered between 0.3 and 45 Hz prior to spectral analysis. Consistent with the preregistration, only N3 delta power (0.5–4 Hz) and standard sleep statistics were retained for subsequent analyses. All sleep variables and continuous variables were z-scored prior to statistical analysis to improve numerical stability during model estimation. Summary sleep statistics are reported in Table S23.

### Quantification and statistical analysis

#### Behavioural data statistical analysis

Behavioural data were pre-processed in Python (v3.12.7) and analyzed in R (v4.4.2). Data were analyzed using ANOVAs, t-tests, mixed-effects models using *rstatix, lmerTest*, *brms* with model assumptions assessed using *performance* with visualizations generated using *ggplot2*, *sjPlot* and *interactions*.^56^^,^^57^^,^^58^^,^^59^^,^^60^^,^^61^ Trait anxiety was modelled both categorically and continuously. Dissipation in state anxiety was defined as the evening-to-morning change in STAI-S scores (pre-post), with positive values indicating reduced morning state anxiety. All measures were z-scored prior to modelling.

Parametric assumptions were assessed prior to analysis; when violated, appropriate non-parametric tests were applied, with rank-based ANOVAs and Holm-corrected pairwise comparisons used where necessary. For mixed-effects models, model selection was performed using Akaike Information Criterion (AIC), with the best-fitting model determined by lowest AIC weight. Bayesian beta regression models were estimated using 4 MCMC chains of 2,000 iterations each (1,000 warmup), yielding 4,000 post-warmup samples. Convergence was confirmed ($\hat{R}$ < 1.05; ESS > 400), and model adequacy was assessed via posterior predictive checks; only well-converged models are reported.

To examine whether N3 sleep uniquely predicted overnight changes in task performance, we fitted a series of linear mixed-effects models predicting post-minus-pre sleep changes in behavioural measures. Prior to model fitting, EEG-derived total sleep time was validated against subjective sleep duration reports, which showed a significant positive correlation (Pearson’s correlation, r(38) = 0.36, *p* <.05). Candidate models included N3 sleep duration, N3 delta-band power, REM latency, and overnight change in state anxiety as predictors, reflecting preregistered hypotheses regarding sleep architecture and anxiety-related influences on learning. Model selection was based on goodness-of-fit (AIC) and diagnostic checks of model assumptions (Table S15).

Descriptive data and frequentist estimates are reported as means ± SEM, unless otherwise indicated. Bayesian parameter estimates are summarized using posterior estimates with 95% credible intervals or highest density intervals (HDIs), with Bayesian effects interpreted according to the specified criteria (Bayes Factor > 1 or q<.05) for each analysis. Exact statistical tests are reported in the corresponding figure legends and Results, with detailed model specifications and comparisons provided in the indicated Supplementary Tables. For figure legends, ✱ denotes statistically significant effects based on frequentist analyses (*p* <.05), whereas ✽ denotes effects based on Bayesian analyses. Experiment 1 included 50 participants, of whom 48 were included in the final analysis after exclusion of two participants. Experiment 2 included 40 participants, of whom 40 were included in the final analysis. Unless otherwise specified, N refers to the number of participants included in the analysis.

#### Computational modelling

Hierarchical estimation yields superior parameter recovery and statistical power relative to non-hierarchical approaches by jointly constraining group- and individual-level distributions.^10^^,^^62^

##### Hierarchical Bayesian reinforcement learning models

Learning rates were estimated using hierarchical Bayesian reinforcement-learning models implemented in hBayesDM in R.^63^ For both experiments, multiple candidate models were fit (Tables S4 and S18) using four Markov chains with 4,000 iterations each (1,000 burn-in). Models were fit to trial-wise patch choices (1–3), gains (smiley emoji: 0/1), and losses (sad emoji: 0/–1), with each participant contributing repeated trial-level observations per block. In Experiment 1, hierarchical priors were specified separately for anxiety group (high/low) and block (six blocks). In Experiment 2, priors were specified separately for session (pre-/post-sleep) and block (four blocks per session). Model convergence and reliability were assessed using $\hat{R}$ (<1.05), effective sample size (ESS and tail-ESS), and Pareto-k diagnostics. Model comparison was based on Leave-One-Out Information Criterion (LOOIC), and the best-fitting model was selected as the one with the lowest LOOIC and no convergence or reliability violations (Tables S4 and S19). Additional model comparisons tested alternative prior structures, including pooled, group-only (anxiety or session), block-only, and group-by-block priors (Tables S5 and S19). In Experiment 1, a five-parameter model with separate reward and punishment learning rates, sensitivities, and a noise parameter provided the best fit. Incorporating both anxiety- and block-specific priors yielded the lowest LOOIC, indicating differential block-wise learning between anxiety groups. In Experiment 2, the best-fitting model additionally included a decay parameter, with session-and-block priors providing the best fit, indicating distinct learning across blocks and sleep sessions.

Model adequacy was evaluated using posterior predictive checks. Choices simulated from the winning model were compared with empirical behaviour by correlating switch probabilities across trials; correlations exceeding 0.7 were taken as evidence of good model fit.^10^ The winning models showed strong correspondence with observed data (Pearson’s correlation, Experiment 1: r(46)=0.92, *p* <.001; Experiment 2: pre-sleep: r(38)=0.86, *p* <.05; post-sleep: r(38)=0.88, *p* <.05). Group-level differences in parameters were assessed using 95% highest density intervals (HDIs) on hyperparameter differences, with effects considered reliable when HDIs did not include zero. In Experiment 1, post hoc analyses of individual-level parameter estimates were conducted using Bayesian mixed models (*brms*) to assess effects of stochasticity, volatility, and trait anxiety, including continuous symptom analyses with STAI-Y scores. In Experiment 2, mixed-effects regressions were conducted on individual-level parameter estimates to quantify sleep-related changes in learning. For each participant and block, the difference between post-sleep and pre-sleep parameter values (e.g., change in reward learning rate) was computed and used as the dependent variable. These difference scores indexed sleep-related modulation of model parameters and were analysed to evaluate the joint effects of sleep (including N3 duration) and anxiety across all blocks.

To identify optimal parameter values that maximized rewards within each block, we simulated behaviour using the best-fitting model across a broad parameter space. For each model parameter, nine values were selected spanning the empirical range of estimates; for learning rates, values ranged from 0.1 to 0.9. This resulted in 59,049 unique parameter combinations. For each parameter set, choices, gains, and losses were simulated across blocks. The top-performing 0.1% of simulated agents (50 agents per block) with the highest total rewards were identified, and their parameter values were averaged to define optimal parameters for that block. Individual deviation scores were then computed as the difference between participants’ parameter estimates and the corresponding optimal values. Deviation scores were evaluated using Bayesian one-sample t-tests against a population mean of zero, with Bayesian correlations and tests conducted using *rstanarm* and *bayestestR*.^64^^,^^65^ Effects were considered meaningful when 95% credible intervals excluded zero, and Bayes factors exceeded 1.

##### Hierarchical drift-diffusion models

Process-level modelling of the task behaviour was performed using hierarchical drift-diffusion modelling was performed using HDDM package (v0.9.8) within a containerized Python environment (Docker), ensuring reproducible software dependencies. The drift-diffusion model comprises of four parameters estimated from trial-wise reaction times (RTs): drift rate (v), reflecting the speed of evidence accumulation towards an option; boundary separation (a), represents how much information is required to be accumulated to make a choice; starting-point bias (z); and non-decision time (t) captures sensory and motor processes unrelated to evidence accumulation.^66^ Trial-wise choices were modeled using two boundaries: selecting the most rewarding patch versus any other patch (1/0), with each participant contributing repeated trial-level observations per block. In Experiment 1, model parameters were allowed to vary as a function of the block and trait anxiety group (high/low). In Experiment 2, parameters varied by block and session (pre-/post-sleep). For each experiment, 10 candidate hierarchical models were fit, differing in prior structure (single pooled, block-only, anxiety/session-only, or anxiety/session×block) and parameter inclusion (full model or reduced models including subsets of parameters). All models were estimated using four chains with 4,000 samples each (1,000 burn-in). Model selection was based on convergence ($\hat{R}$ < 1.05) and Deviance Information Criterion (DIC), with the lowest DIC identifying the winning model.

In Experiment 1, the best-fitting model included block- and anxiety-dependent effects on drift rate and boundary separation (Table S9). In Experiment 2, the winning model included session- and block-dependent effects on drift rate, boundary separation, and starting-point bias, with non-decision time varying by session only (Table S21). Model adequacy was assessed using posterior predictive checks. For each winning model, 500 datasets were simulated using parameters sampled from participants’ posterior distributions (300 trials per simulation). Summary statistics from the empirical data fell within the 95% credible intervals of the simulated data, indicating good model fit. Convergence and reliability were further confirmed using effective sample size (ESS) diagnostics. Group-level differences were assessed by comparing posterior distributions of parameters, with <5% overlap taken as evidence of reliable differences^67^^,^^68^ (q<.05). In Experiment 2, sleep-related change scores (post–pre) derived from individual parameter estimates i.e, Δdrift rate were analyzed using mixed-effects regressions to test effects of sleep and trait anxiety.

### Additional resources

We pre-registered both experiments on AsPredicted.

Experiment I: https://aspredicted.org/cr8p7i.pdf.

Experiment 2: https://aspredicted.org/vy89xw.pdf.
