# Supplementary material for: Trait anxiety selectively amplifies reward learning under uncertainty, while N3 sleep attenuates it
Source: iScience. 2026 Oct 1;29(10):117745. doi: 10.1016/j.isci.2026.117745 (PMC13657353; doi:10.1016/j.isci.2026.117745)
Supplement: Supplementary file 1 [file mmc1.pdf]

iScience, Volume *29*

Supplemental information

Trait anxiety selectively amplifies reward learning under uncertainty, while N3 sleep attenuates it

Rakshita Deshmukh and Arjun Ramakrishnan

Figures

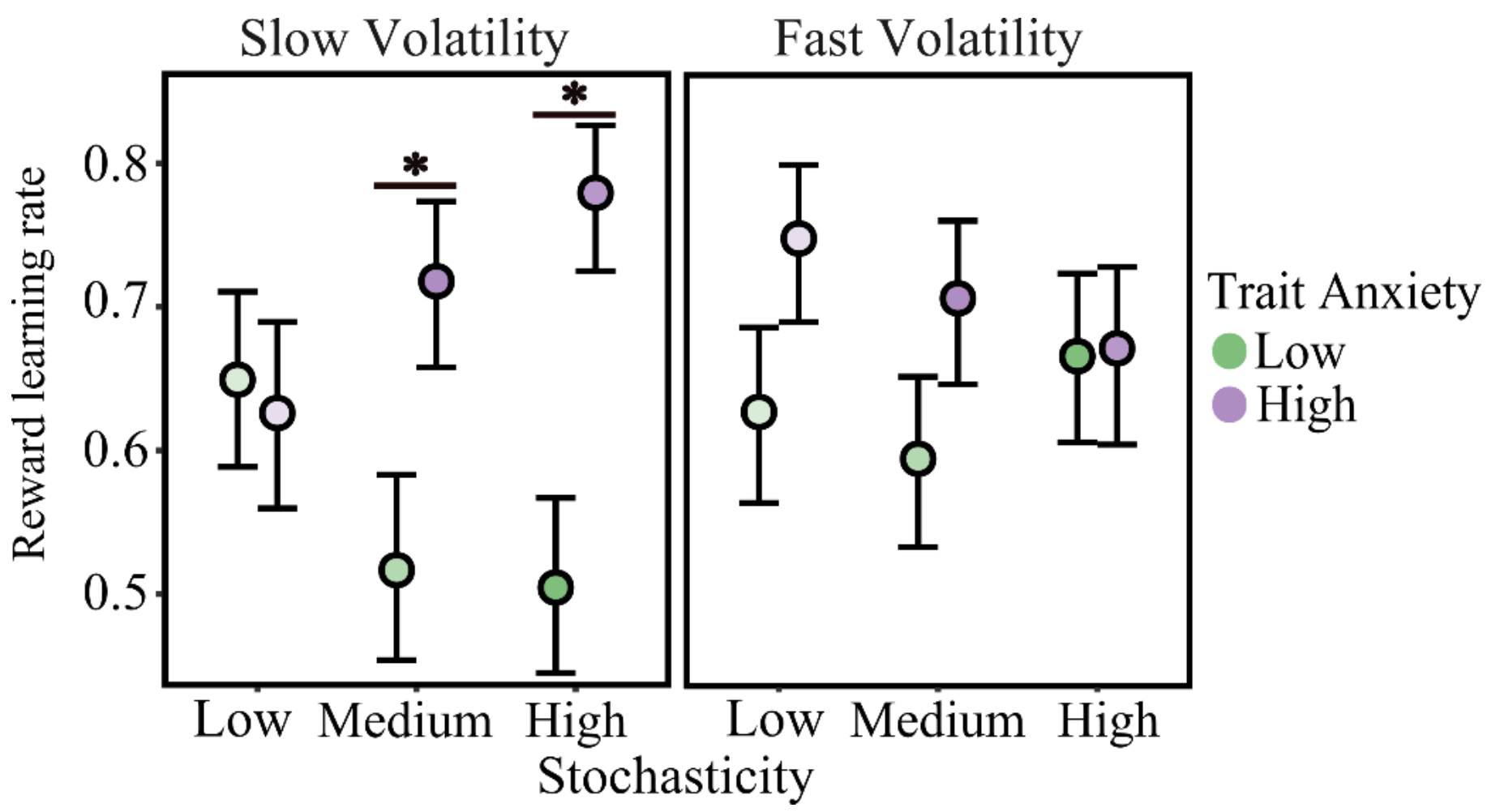

Figure S1. High trait anxiety and reward learning rates in slow-volatility, high-stochasticity environments (related to Figure 2). Posterior means (± SEM) of reward learning rates show that when trait anxiety was treated categorically (HTA; STAI-T ≥ 45), reward learning rates did not differ between anxiety groups in fast-volatility environments. In contrast, under slow volatility, HTA individuals exhibited higher reward learning rates than LTA individuals as stochasticity increased. Note: Asterisks (✻) indicate statistically credible effects based on Bayesian inference. Data are shown for 48 participants; two participants were excluded from this analysis per exclusion criteria (See STAR★METHODS).

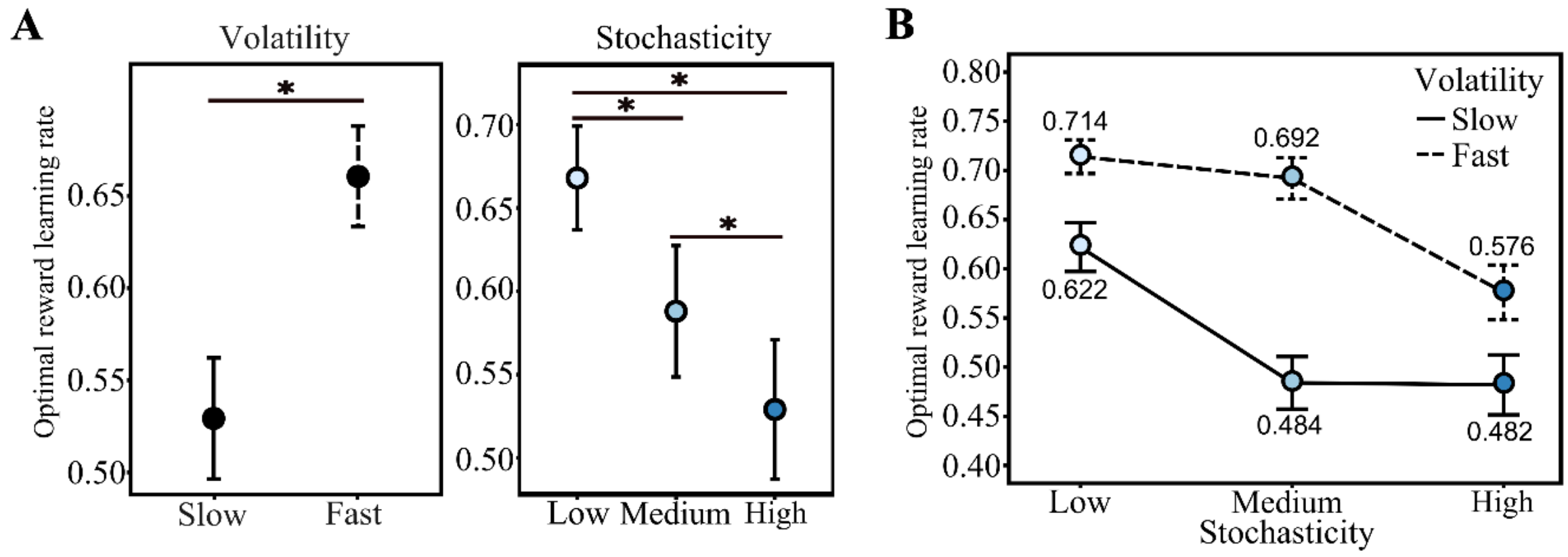

Figure S2 Evidence #2 Optimal reward learning rates across environments (related to Figure 2 & STAR Methods). (A) Higher volatility favors higher learning, while higher stochasticity favors lower learning for maximizing rewards as expected. Two-way ANOVA show main effect of stochasticity and volatility (Stochasticity: $F_{2,294}=15.49$, $p<.001$, $\eta^2_p=0.10$; Volatility: $F_{1,294}=41.18$, $p<.001$, $\eta^2_p=0.12$), where higher levels of stochasticity lower reward learning rates, while fast volatility increases reward learning rates (Post-hoc t-tests, Stochasticity Low>Medium>High: Low>Medium: $t_{100}=3.16$, $p<.01$, $d=0.33$, 95%CI [0.03, 0.13]; Low>High: $t_{100}=5.29$, $p<.001$, $d=0.52$, 95%CI [0.09, 0.19]; Medium>High: $t_{100}=2.04$, $p<.05$, $d=0.20$, 95%CI [0.002, 0.12]; Volatility Fast>Slow: $t_{150}=6.08$, $p<.001$, $d=0.70$, 95%CI [0.09, 0.17]). **(B)** Optimal reward learning rate value for each environment. We observed a weak interaction between volatility and stochasticity which is expected when the source of uncertainty is ambiguous ($F_{2,294}=3.51$, $p<.05$, $\eta^2_p=0.02$). Both simple main effects of volatility on stochasticity (Slow: $F_{2,147}=10.90$, $p<.001, \eta^2_p=0.13$; Fast: $F_{2,147}=8.57$, $p<.001, \eta^2_p=0.10$) and of stochasticity on volatility (Low: $F_{1,98}=9.32$, $p<.01, \eta^2_p=0.087$; Medium; $F_{1,98}=37.53$, $p<.001, \eta^2_p=0.28$; High: $F_{1,98}=6.17$, $p<.01, \eta^2_p=0.05$) were significant, where across all levels of volatility, high stochasticity lowered reward learning rates (Post-hoc t-tests, Slow volatility: Low>Medium: $t_{98}=3.79$, $p<.001$, $d=0.76$, 95%CI [0.07, 0.21]; Low>High: $t_{94}=3.56$, $p<.001$, $d=0.71$, 95%CI [0.06, 0.22]; Medium>High: $t_{96}=0.05$, $p=.9$, $d=0.01$, 95%CI [-0.08, 0.08]; Fast volatility: Low>Medium: $t_{94}=0.81$, $p=.4$, $d=0.16$, 95%CI [-0.03, 0.08]; Low>High: $t_{81}=4.21$, $p<.001$, $d=0.84$, 95%CI [0.07, 0.20]; Medium>High: $t_{91}=3.32$, $p<.01$, $d=0.66$, 95%CI [0.05, 0.19]), and across all levels of stochasticity, fast volatility increased reward learning rates (Post-hoc t-tests, Low stochasticity: Fast>Slow: $t_{87}=3.05$, $p<.001$, $d=0.61$, 95%CI [0.03, 0.15]; Medium stochasticity: Fast>Slow: $t_{87}=6.13$, $p<.001$, $d=1.23$, 95%CI [0.14, 0.28]; High stochasticity: Fast>Slow: $t_{87}=2.27$, $p<.05$, $d=0.45$, 95%CI [0.01, 0.18]). Although two pairwise comparisons were non-significant (under slow volatility, Medium vs. High stochasticity, $p=.90$; and under fast volatility, Low vs. Medium stochasticity, $p=.40$), the overall expected pattern was observed, with reward learning rates generally decreasing as stochasticity increased. *Note*: Asterisks (∗) denote significant effects based on frequentist statistics. Data are shown for top-performing 0.1% of simulated agents (N=59, see STAR★METHODS).

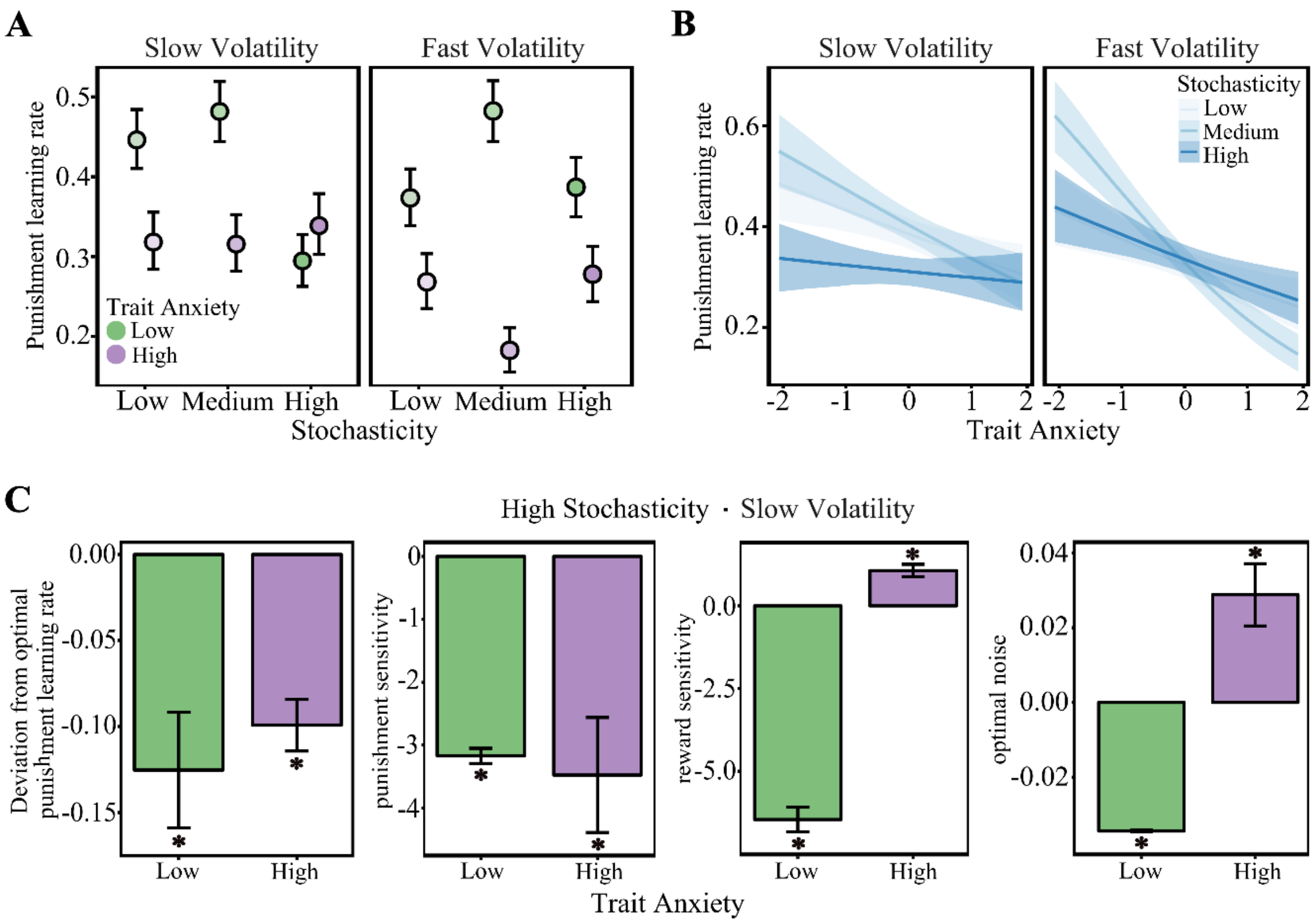

Figure S3. Effect of trait anxiety on punishment learning rates (related to Figure 2). Punishment learning rates from Bayesian beta mixed regression models**.** **(A)** Posterior means (±SEM) of punishment learning rates for high- and low-trait-anxious (HTA, LTA) participants across volatility and stochasticity conditions. **(B)** Posterior estimates showing the relationship between continuous trait anxiety and punishment learning rates across volatility and stochasticity conditions. Both categorical and continuous trait anxiety scores showed lower punishment learning rates than LTA counterparts. Based on the group-level hyperparameter estimates: HTA lower punishment learning rate than LTA in medium stochasticity-fast volatility environment (95% HDI [1.52, 9.91]). In slow-volatility, high-stochasticity environment, rates were nearly identical across groups, suggesting that punishment learning did not drive performance in this environment. **(C)** Comparison of trait anxiety groups with task-optimal parameters (excluding reward learning rate) in the high stochasticity–slow volatility environment. Both HTA and LTA participants deviated from optimal values across these parameters. Punishment learning rates and punishment sensitivity were lower than optimal in both groups, indicating that punishment-related processes did not contribute to reward maximization (Bayesian one-sample tests, Punishment learning rate: HTA: β=-0.10, 95%CI [-0.13, -0.07], BF=12100; LTA: β=-0.13, 95%CI [-0.21, -0.06], BF=34.41; Punishment sensitivity: HTA: β=-3.74, 95% CI [-5.70, -1.79], BF = 34.49; LTA: β=-3.18, 95% CI [-3.42, -2.93], BF = 4.46e+16). In contrast, HTA participants exhibited higher reward sensitivity and greater noise (randomness in responding) than optimal, whereas LTA participants showed lower reward sensitivity and noise (Bayesian one-sample tests, Reward Sensitivity: HTA: β=1.07, 95%CI [0.66, 1.45], BF = 1740; LTA: β=-6.50, 95% CI [-7.28, -5.72], BF = 3.09e+12; Noise: HTA: β=0.03, 95% CI [0.01, 0.04], BF = 17.08; LTA: β=-0.03, 95% CI [-0.03, 0.03], BF= 7.92e+35). Together with their elevated reward learning rates, these findings suggest that HTA individuals adopted a distinct reward-focused strategy characterized by heightened reward sensitivity and increased decision noise. Notably, both groups showed reduced punishment learning rates and sensitivity relative to optimal values, indicating that differences in punishment-related parameters did not account for performance differences. Instead, reward-focused parameters primarily drove the reduced performance observed in HTA. Data are shown for 48 participants; two participants were excluded from this analysis per exclusion criteria (See STAR★METHODS).

Tables

Table S1. Mixed effects ANOVA table on total rewards (Related to STAR Methods).
Within-subject variables: stochasticity and volatility Between-subject variable: categorical trait anxiety (High trait anxious; STAI-T score ≥ 45). Significant differences are highlighted in bold where p<.05.

**Predictor**		df_{num}	df_{den}	**F**	***p***	η^2_p
Stochasticity		2	276	74.164	**.000**	0.350
Volatility		1	276	7.692	**.006**	0.027
Trait anxiety		1	276	4.076	**.044**	0.015
Stochasticity x Volatility		2	276	0.472	.624	0.003
Stochasticity x Trait anxiety		2	276	0.538	.585	0.004
Volatility x Trait anxiety		1	276	4.866	**.028**	0.017
Stochasticity x Volatility x Trait anxiety		2	276	1.093	.337	0.008
**Post-hoc pairwise comparisons**			**df**	**t**	***p***	*d*
Stochasticity	Low > Medium		95	7.74	**.000**	0.79
	Low > High		95	13.67	**.000**	1.39
	Medium > High		95	4.88	**.000**	0.5
Volatility	Slow > Fast		144	3.00	**.003**	0.25
Trait Anxiety	Slow volatility: HTA < LTA		129	-2.3	**.023**	-0.33
	Fast volatility: HTA < LTA		129	0.11	.911	0.03

Note: df_{num} indicates degrees of freedom numerator, df_{den} indicates degrees of freedom denominator, η^2_p indicates partial eta-squared values i.e., the effect size, and *d* indicates Cohen's d effect size.

Table S2. Between subject t-tests for every block/environment between HTA (group1) and LTA (group 2) individuals on total rewards (Related to STAR Methods).

To examine environment specific effects on total rewards. Significant differences are highlighted in bold where $p<.05$.

Block	df	t	*p*	95% CI	*d*
Low Stochasticity- Slow Volatility	41.05	-0.53	.598	-4.36, 2.54	-0.15
Medium Stochasticity- Slow Volatility	44.11	-1.31	.197	-5.31, 1.12	-0.38
High Stochasticity- Slow Volatility	36.78	-2.61	**.013**	-6.32, -0.79	-0.77
Low Stochasticity- Fast Volatility	45.27	0.32	.749	-2.48, 3.43	0.09
Medium Stochasticity- Fast Volatility	44.63	-0.44	.660	-2.81, 1.79	-0.13
High Stochasticity- Fast Volatility	39.05	0.4	.695	-1.91, 2.84	0.12

Note: df indicates degrees of freedom, 95% CI indicates 95% confidence interval, and *d* indicates effect size using Cohen's *d*.

Table S3. Effect on trialwise learning of the most rewarding patch (Related to STAR Methods). *Zero-one inflated beta (zoib) regression (fixed effects: stochasticity, volatility, trait anxiety; random effects: participant & trial) results in trial-wise learning of the most-rewarding patch. Model fitted with default log links for precision and logit for the mean, one-inflated and zero-inflated functions. Significant differences are highlighted (in bold) when 95% CI does not encompass zero.*

	Estimate (β)	SE	95% CI	
Mean function				
Intercept	0.86	0.06	0.75	0.97
Medium stochasticity	-0.49	0.02	**-0.53**	**-0.44**
High Stochasticity	-0.57	0.02	**-0.62**	**-0.53**
Fast volatility	-0.29	0.02	**-0.33**	**-0.24**
HTA	-0.17	0.08	**-0.32**	**-0.01**
Medium stochasticity x Fast volatility	0.05	0.03	-0.01	0.11
High stochasticity x Fast volatility	-0.1	0.03	**-0.16**	**-0.04**
Medium stochasticity x HTA	-0.12	0.04	**-0.18**	**-0.05**
High stochasticity x HTA	-0.19	0.03	**-0.25**	**-0.13**
Fast volatility x HTA	0.11	0.03	**0.04**	**0.18**
Medium stochasticity x Fast volatility x HTA	0.12	0.05	**0.03**	**0.22**
High stochasticity x Fast volatility x HTA	0.19	0.04	**0.11**	**0.28**
Precision function (phi)				
Intercept	2.45	0.13	2.19	2.7
Medium stochasticity	-0.15	0.06	**-0.27**	**-0.02**
High Stochasticity	0.07	0.06	-0.06	0.19
Fast volatility	0.29	0.07	**0.15**	**0.42**
HTA	0.2	0.18	-0.18	0.54
Medium stochasticity x Fast volatility	0.25	0.09	**0.07**	**0.42**
High stochasticity x Fast volatility	0	0.09	-0.17	0.18
Medium stochasticity x HTA	-0.06	0.1	-0.25	0.13
High stochasticity x HTA	0.12	0.09	-0.06	0.31
Fast volatility x HTA	-0.43	0.1	**-0.63**	**-0.24**
Medium stochasticity x Fast volatility x HTA	-0.33	0.14	**-0.61**	**-0.05**
High stochasticity x Fast volatility x HTA	-0.02	0.13	-0.28	0.23
One-inflated function (coi)				

Intercept	3.64	0.74	2.26	5.17
Medium stochasticity	-0.75	0.44	-1.61	0.11
High Stochasticity	-1.87	0.46	**-2.77**	**-0.97**
Fast volatility	0.11	0.47	-0.81	1.03
HTA	-0.38	0.89	-2.11	1.35
Medium stochasticity x Fast volatility	-0.67	0.65	-2.01	0.6
High stochasticity x Fast volatility	0.76	0.67	-0.61	2.02
Medium stochasticity x HTA	-0.47	0.72	-1.88	0.97
High stochasticity x HTA	-0.03	0.74	-1.45	1.45
Fast volatility x HTA	-1.14	0.69	-2.48	0.24
Medium stochasticity x Fast volatility x HTA	1.9	0.97	-0.03	3.77
High stochasticity x Fast volatility x HTA	1.47	1.02	-0.53	3.45
Zero-inflated function (zoi)				
Intercept	-4.87	0.87	-6.69	-3.27
Medium stochasticity	-1.08	0.19	**-1.46**	**-0.7**
High Stochasticity	-1.17	0.19	**-1.55**	**-0.8**
Fast volatility	-1.26	0.2	**-1.64**	**-0.87**
HTA	-0.45	0.43	-1.31	0.4
Medium stochasticity x Fast volatility	0.52	0.3	-0.05	1.1
High stochasticity x Fast volatility	0.4	0.3	-0.19	0.97
Medium stochasticity x HTA	-0.61	0.31	**-1.22**	**-0.01**
High stochasticity x HTA	-0.99	0.32	**-1.63**	**-0.38**
Fast volatility x HTA	0.58	0.29	**0.01**	**1.12**
Medium stochasticity x Fast volatility x HTA	0.87	0.45	**0.02**	**1.74**
High stochasticity x Fast volatility x HTA	1.06	0.45	**0.16**	**1.93**

Table S4. Hierarchical reinforcement learning bayesian analysis model comparison (Related to STAR Methods).

Model Comparisons with model specifications and LOOIC values. Highlighting (in bold) the best model with lowest LOOIC score.

Model	#Parameters	Parameters	LOOIC
banditNarm_delta	2	Learning rate<br>Inverse temperature	20312.99
banditNarm_2par_lapse	3	Reward learning rate<br>Punishment learning rate<br>Lapse	25109.97
banditNarm_4par	4	Reward learning rate<br>Punishment learning rate<br>Reward sensitivity<br>Punishment sensitivity	17856.41
banditNarm_lapse_decay	6	Reward learning rate<br>Punishment learning rate<br>Reward sensitivity<br>Punishment sensitivity<br>Lapse<br>Decay rate	18012.77
banditNarm_singleA_lapse	4	Learning rate<br>Reward sensitivity<br>Punishment sensitivity<br>Lapse	18235.81
banditNarm_lapse	5	Reward learning rate<br>Punishment learning rate<br>Reward sensitivity<br>Punishment sensitivity<br>Lapse	**17851.88**

Table S5. Winning model prior fits (Related to STAR Methods).
Model prior combinations of the best model. Selecting the best model with the best prior fits which have the lowest LOOIC values without any model convergence/reliability issues. [x]Denotes that the model did not converge, making the LOOIC values for the prior meaningless.

**Priors**	**LOOIC**
	banditNarm_lapse
Anxiety and Block Priors (12)	**17851.88**
Anxiety Priors (2)	18379.17
Block Priors (6)	17755.31[x]
Single Prior (1)	18354.88[x]

Table S6. Parameter estimates and group comparison on the winning model and prior combination (Related to STAR Methods).

Comparing parameter estimates of the best model between HTA v/s LTA for each block. In the HTA and LTA columns, the values represent the mean (standard deviation) of the average posterior estimates obtained from each individual. The 'between group HDI' column represents the lower and upper bounds of the 95% HDI. The intervals that do not encompass zero have been highlighted (in bold) as these are meaningful differences between the two groups. HDIs have been computed by taking the difference between HTA and LTA (HTA - LTA) for all parameters per block.

banditNarm_lapse	Block	HTA	LTA	Between group HDI
Reward learning rate	80slow	0.63 (0.03)	0.65 (0.01)	-0.18 0.17
	70slow	0.72 (0.02)	0.52 (0.03)	**0.03 0.39**
	60slow	0.75 (0.04)	0.51 (0.04)	**0.11 0.51**
	80fast	0.75 (0.01)	0.63 (0.02)	-0.05 0.29
	70fast	0.70 (0.03)	0.60 (0.03)	-0.07 -0.33
	60fast	0.66 (0.05)	0.67 (0.01)	-0.19 0.21
Punishment learning rate	80slow	0.32 (0.02)	0.45 (0.00)	-0.41 0.11
	70slow	0.31 (0.01)	0.48 (0.01)	-0.35 0.02
	60slow	0.34 (0.02)	0.31 (0.03)	-0.21 0.26
	80fast	0.27 (0.03)	0.37 (0.01)	-0.27 0.06
	70fast	0.19 (0.02)	0.48 (0.01)	**-0.51 -0.12**
	60fast	0.28 (0.01)	0.39 (0.01)	-0.27 0.04
Reward sensitivity	80slow	7.98 (0.69)	7.08 (0.65)	-2.65 4.67
	70slow	5.70 (0.62)	6.31 (0.45)	-3.73 1.72
	60slow	16.43 (0.19)	8.90 (0.37)	-1.81 18.12
	80fast	7.06 (0.61)	7.79 (0.57)	-5.1 3.48
	70fast	11.08 (0.61)	8.61 (0.56)	-3.15 9.38
	60fast	10.14 (0.76)	7.08 (0.81)	-2.45 10.64
Punishment sensitivity	80slow	4.00 (0.31)	2.24 (0.21)	-1.24 4.71
	70slow	3.52 (0.54)	3.12 (0.41)	-1.9 2.62
	60slow	5.06 (0.91)	5.37 (0.12)	-5.95 3.72
	80fast	5.47 (0.27)	5.83 (0.68)	-3.51 3.6

	70fast	8.20 (0.64)	3.07 (0.57)	**1.52 9.91**
	60fast	4.98 (0.81)	3.75 (0.61)	-2.03 3.94
Lapse	80slow	0.02 (0.00)	0.01 (0.00)	-0.03 0.03
	70slow	0.02 (0.00)	0.02 (0.00)	-0.04 0.05
	60slow	0.08 (0.01)	0.01 (0.00)	-0.003 0.11
	80fast	0.02 (0.00)	0.03 (0.00)	-0.05 0.04
	70fast	0.02 (0.00)	0.02 (0.00)	-0.03 0.04
	60fast	0.03 (0.00)	0.02 (0.00)	-0.04 0.06

Table S7. Effect on reward learning rates (Related to STAR Methods).
Reward learning rate results using bayesian beta regression for when trait anxiety was categorical and on a continuous scale. Significant differences are highlighted (in bold) when 95% CI does not encompass zero.

Fixed effects	*Categorical Trait Anxiety*				*Continuous Trait Anxiety*			
	**β**	**SE**	**95% CI**		**β**	**SE**	**95% CI**	
Intercept	0.62	0.14	**0.36**	**0.9**	0.57	0.1	0.38	0.76
Medium Stochasticity	-0.55	0.15	**-0.85**	**-0.26**	-0.11	0.11	-0.33	0.11
High Stochasticity	-0.6	0.15	**-0.88**	**-0.32**	0.00	0.11	-0.22	0.23
Fast Volatility	-0.1	0.15	-0.39	0.2	0.2	0.11	-0.04	0.41
HTA	-0.1	0.2	-0.5	0.28	0.07	0.09	-0.12	0.25
Medium Stochasticity:Fast Volatility	0.41	0.21	-0.01	0.82	-0.06	0.16	-0.37	0.26
High Stochasticity:Fast Volatility	0.77	0.21	**0.36**	**1.18**	-0.08	0.16	-0.39	0.24
Medium Stochasticity:HTA	0.97	0.22	**0.53**	**1.42**	0.36	0.12	**0.13**	**0.59**
High Stochasticity:HTA	1.34	0.23	**0.9**	**1.79**	0.62	0.12	**0.39**	**0.85**
Fast Volatility:HTA	0.67	0.23	**0.21**	**1.11**	0.17	0.12	-0.06	0.4
Medium Stochasticity:Fast Volatility:HTA	-1.04	0.32	**-1.67**	**-0.41**	-0.41	0.16	**-0.73**	**-0.1**
High Stochasticity:Fast Volatility:HTA	-1.89	0.32	**-2.53**	**-1.25**	-0.83	0.17	**-1.16**	**-0.5**
**Random effects**								
Intercept: participant	0.38	0.06	0.28	0.51	0.37	0.06	0.27	0.49
**Family specific parameters**								
Precision function	14.11	1.28	11.71	16.79	13.90	1.25	11.56	16.47

Table S8. Evidence #1 Comparing learning rates with rewards earned (Related to STAR Methods).

Comparing relationship between reward and punishment learning rates and total rewards earned in every block using bayesian correlations. Significant differences (when %ROPE is less than 1) are highlighted (in bold).

Parameter	Block	Median	95% CI	Probability of Direction (pd)	%ROPE	Bayes factor (BF)
Reward learning rate	80slow	-0.05	-0.32, 0.20	65.50%	28.03%	0.348
	70slow	-0.23	-0.47, 0.03	95.73%	6.79%	1.52
	60slow	-0.36	-0.57, -0.09	99.35%	**0%**	10.80
	80fast	-0.01	-0.27, 0.27	52.20%	28.29%	0.325
	70fast	0.27	-0.01, 0.50	97.30%	3.61%	2.11
	60fast	0.19	-0.07, 0.44	92.95%	10.47%	0.863
Punishment learning rate	80slow	-0.03	-0.28, 0.25	56.85%	28.89%	0.328
	70slow	0.11	-0.16, 0.37	79.22%	22.18%	0.449
	60slow	-0.12	-0.37, 0.17	81.15%	20.53%	0.473
	80fast	-0.13	-0.38, 0.14	82.83%	19.71%	0.505
	70fast	0.05	-0.21, 0.32	63.98%	28.95%	0.351
	60fast	-0.16	-0.41, 0.12	87.90%	14.92%	0.674

Table S9. Evidence #3 Hierarchical drift-diffusion model comparisons (Related to STAR Methods).

Model Comparisons with model specifications and DIC values. Highlighting (in bold) the best model i.e., with the lowest DIC score. Depends_on column denotes which parameters were fit on which priors. [x]Denotes that the model did not converge (R-hat value greater than 1.05), making the DIC values for the prior meaningless.

Priors	Parameters depends_on				DIC
Anxiety * Block	*v* : anxiety, block	*a* : anxiety, block	*t* : anxiety, block	*z* : anxiety, block	40076.67[x]
Anxiety*Block	*v* : anxiety, block	*a* : anxiety, block	*t* : anxiety	*z* : anxiety	**42040.86**
Anxiety*Block	*v* : anxiety, block	*a* : anxiety, block	*t* : anxiety	*z* : anxiety, block	43379.99
Anxiety*Block	*v* : anxiety, block	*a* : anxiety	*t* : anxiety	*z* : anxiety	45524.81
Block	*v* : block	*a* : block	*t* : block	*z* : block	41417.28[x]
Block	*v* : block	*a* : block	*t*	*z* : block	43376.46
Block	*v* : block	*a* : block	*t*	*z*	43519.07
Block	*v* : block	*a*	*t*	*z*	45519.17
Anxiety	*v* : anxiety	*a* : anxiety	*t* : anxiety	*z* : anxiety	46272.20
Single	*v*	*a*	*t*	*z*	46271.60

Table S10. Evidence #3 Parameters estimates of drift-diffusion model (Related to STAR Methods).

Comparing parameter estimates of the best model between HTA v/s LTA for each block by using their posterior distributions. In the HTA and LTA columns, the values represent the mean (standard deviation) of the average posterior estimates obtained from each individual

**Parameter**	**Block**	**HTA**	**LTA**	**Direction of comparison**	**% difference**	***q***
Drift-rate (*v*)	80slow	0.51 (0.07)	0.58 (0.06)	HTA < LTA	76.93%	0.231
	70slow	0.08 (0.07)	0.29 (0.06)		98.97%	**0.010**
	60slow	0.03 (0.07)	0.19 (0.06)		94.84%	**0.052**
	80fast	0.25 (0.07)	0.26 (0.06)		55.04%	0.450
	70fast	0.07 (0.07)	0.09 (0.06)		54.81%	0.452
	60fast	-0.09 (0.07)	-0.04 (0.06)		71.06%	0.289
Boundary / threshold (*a*)	80slow	1.84 (0.09)	1.93 (0.08)	HTA > LTA	22.66%	0.773
	70slow	2.11 (0.09)	2.11 (0.08)		51.32%	0.487
	60slow	1.99 (0.09)	1.79 (0.08)		95.08%	**0.049**
	80fast	2.12 (0.09)	2.11 (0.08)		52.74%	0.473
	70fast	1.77 (0.09)	1.80 (0.08)		42.01%	0.579
	60fast	2.06 (0.09)	1.88 (0.08)		92.10%	0.079
Non-decision time (*t*)	Overall	1.92 (0.06)	1.96 (0.05)	HTA < LTA	70.99%	0.290
Bias (*z*)	Overall	0.01 (0.02)	-0.04 (0.02)	HTA > LTA	92.24%	0.078

Note: % difference indicates the percentage of the difference between the two posterior distributions, q indicates the q-value i.e., percentage overlap with significant differences (<0.05) highlighted (in bold). Drift-rate 60slow q-value is near significance which is also highlighted.

Table S11. Effect on punishment learning rates (Related to STAR Methods).
Punishment learning rate results using bayesian beta regression for when trait anxiety was categorical and on a continuous scale.

**Fixed effects**	***Categorical Trait Anxiety***				***Continuous Trait Anxiety***			
	**β**	**SE**	**95% CI**		**β**	**SE**	**95% CI**	
Intercept	-0.22	0.08	**-0.36**	**-0.06**	-0.47	0.06	**-0.59**	**-0.34**
Medium Stochasticity	0.14	0.08	-0.02	0.3	0.07	0.07	-0.07	0.21
High Stochasticity	-0.66	0.09	**-0.83**	**-0.49**	-0.33	0.07	**-0.48**	**-0.19**
Fast Volatility	-0.3	0.08	**-0.46**	**-0.14**	-0.27	0.07	**-0.41**	**-0.13**
HTA	-0.55	0.12	**-0.77**	**-0.32**	-0.2	0.06	**-0.32**	**-0.07**
Medium Stochasticity:Fast Volatility	0.3	0.12	**0.07**	**0.53**	-0.05	0.11	-0.26	0.16
High Stochasticity:Fast Volatility	0.71	0.12	**0.48**	**0.95**	0.38	0.11	**0.18**	**0.59**
Medium Stochasticity:HTA	-0.15	0.13	-0.4	0.09	-0.09	0.08	-0.24	0.06
High Stochasticity:HTA	0.75	0.13	**0.5**	**0.99**	0.14	0.08	-0.01	0.29
Fast Volatility:HTA	0.06	0.13	-0.19	0.31	-0.03	0.08	-0.18	0.12
Medium Stochasticity:Fast Volatility:HTA	-0.79	0.19	**-1.15**	**-0.42**	-0.27	0.11	**-0.47**	**-0.05**
High Stochasticity:Fast Volatility:HTA	-0.76	0.18	**-1.11**	**-0.4**	-0.13	0.11	-0.34	0.09
**Random effects**								
Intercept: participant	0.24	0.03	0.19	0.32	0.27	0.04	0.20	0.35
**Family specific parameters**								
Precision function	45.21	4.18	37.32	53.51	32.91	3.09	26.92	39.37

Table S12. Comparing all sleep stages dissipation of state anxiety (Related to STAR Methods). *Pearson's correlation between sleep stages and dissipation of state anxiety (pre-post sleep state). Only N3 sleep is significantly correlating with change in morning anxiety.*

**Sleep Stage**	**r**	**df**	***p***
N1	0.17	38	0.30
N2	0.03	38	0.84
N3	**0.36**	38	**0.02**
REM	0.21	38	0.20

Table S13. Effect of N3 sleep on dissipation in state anxiety (Related to STAR Methods). *Linear regression results where dissipation in state anxiety was the dependent variable, with trait anxiety, state anxiety and SCI as independent variables.*

**Predictors**	**β**	**95% CI**	**SE**	**t**	***p***
Intercept	-0.03	-0.32, 0.27	0.14	-0.18	.860
N3	0.46	0.15, 0.77	0.15	3.01	**.005**
Trait Anxiety	-0.23	-0.54, 0.08	0.15	-1.52	.139
SCI	-0.31	-0.63, 0.07	0.16	-1.99	.055
N3:Trait Anxiety	0.43	0.06, 0.79	0.18	2.39	**.023**
N3:SCI	-0.0007	-0.37, 0.37	0.18	-0.004	.997
Trait Anxiety:SCI	-0.03	-0.41, 0.36	0.19	-0.14	.893
N3:Trait Anxiety:SCI	-0.13	-0.66, 0.39	0.26	-0.53	.599

Table S14. Repeated-measures ANOVA table on total rewards (Related to STAR Methods). *Within-subject variables: stochasticity, volatility and session. Significant differences are highlighted in bold where p<.05.*

**Predictor**		df_{num}	df_{den}	**F**	***p***	η^2_p
Stochasticity		1	312	143.86	**.000**	0.316
Volatility		1	312	8.64	**.004**	0.027
Session		1	312	8.91	**.003**	0.028
Stochasticity:Volatility		1	312	5.05	**.025**	0.016
Stochasticity:Session		1	312	0.25	.615	0.001
Volatility:Session		1	312	0.21	.647	0.001
Stochasticity:Volatility:Session		1	312	0.05	.832	0.000
**Post-hoc pairwise comparisons**			**df**	**t**	***p***	*d*
Stochasticity	Low > High		159	6.71	**.000**	0.94
Volatility	Slow > Fast		159	3.27	**.001**	0.26
Session	Post > Pre		159	3.33	**.001**	0.26
Stochasticity:Volatility	Low Stochasticity: Slow > Fast		79	3.66	**.000**	0.41
	High Stochasticity: Slow > Fast		79	-0.785	.513	0.07
	Slow Volatility: Low > High		79	7.96	**.000**	0.96
	Fast Volatility: Low > High		79	5.45	**.000**	0.96

Note: df_{num} indicates degrees of freedom numerator, df_{den} indicates degrees of freedom denominator, η^2_p indicates partial eta-squared values i.e., the effect size, and *d* indicates Cohen's d effect size. Here, high stochasticity and fast volatility lowered rewards but stochasticity and volatility also significantly interacted with each other. Effect of volatility was seen only in low stochasticity condition where fast volatility lowered rewards than slow volatility. While effect of stochasticity was seen in both volatility conditions where high stochasticity lowered rewards than low stochasticity. Thus, higher stochasticity reduced reward accumulation under both slow and fast volatility, whereas changes in volatility had no additional effect when stochasticity was high.

Table S15. Linear mixed-effect model comparison for sleep analysis (Related to STAR Methods).

Model comparisons using AIC values to assess which variable combination is better at predicting the increase in post-sleep rewards. We used a combination of predictors: N3 , REM Latency, Trait Anxiety , DiffState Anxiety , Delta N3 , Stochasticity , Volatility. The best model with lowest AIC value is highlighted in bold.

Predictors	AIC
All variables but only main effects	505.80
All variables interaction i.e., 7th order	509.80
Without stochasticity & volatility interaction, 6th order	516.12
Without Delta N3, ie., 5th order	503.28
4th order interaction	491.5
3rd order interaction, without stochasticity and volatility interaction	**470.9**
3rd order interaction but with stochasticity and volatility interactions	472.76

Table S16. Effect of N3 sleep on increase in post-sleep rewards (Related to STAR Methods). *Linear mixed model (fixed effects: N3 time, REM Latency, Stochasticity, Volatility, Trait Anxiety, Morning State Anxiety; random effect: participant) results on increase in post-sleep rewards.*

**Fixed effects**	**β**	**SE**	**95% CI**	***df***	***t***	***p***
Intercept	-0.1	0.13	-0.36 0.16	140.91	-0.78	0.435
N3	-0.09	0.16	-0.4 0.22	148.74	-0.58	0.561
Trait Anxiety	-0.39	0.16	-0.7 -0.09	141.41	-2.53	**0.013**
Dissipation in State Anxiety	0.05	0.17	-0.28 0.38	117.41	0.3	0.764
REM Latency	-0.1	0.14	-0.38 0.18	147.63	-0.69	0.489
High Stochasticity	-0.05	0.14	-0.33 0.23	120	-0.32	0.747
Fast Volatility	0.11	0.14	-0.17 0.4	120	0.8	0.424
N3:Trait Anxiety	0.46	0.21	0.03 0.88	133.43	2.13	**0.035**
N3:REM Latency	-0.38	0.17	-0.71 -0.05	143.87	-2.28	**0.024**
N3:Dissipation in State Anxiety	0.08	0.08	-0.09 0.25	40	0.95	0.35
N3:High Stochasticity	0.19	0.18	-0.15 0.54	120	1.1	0.273
N3:Fast Volatility	-0.07	0.18	-0.42 0.27	120	-0.42	0.676
Trait Anxiety:REM Latency	0.22	0.11	0.01 0.44	149.02	2.04	**0.044**
Trait Anxiety:Dissipation in State Anxiety	-0.17	0.1	-0.36 0.02	40	-1.79	0.081
Trait Anxiety:High Stochasticity	0.13	0.17	-0.2 0.47	120	0.78	0.437
Trait Anxiety:Fast Volatility	0.04	0.17	-0.29 0.38	120	0.27	0.791
Dissipation in State Anxiety:High Stochasticity	-0.26	0.16	-0.58 0.07	120	-1.57	0.119
Dissipation in State Anxiety:Fast Volatility	0.2	0.16	-0.12 0.53	120	1.23	0.22
REM Latency:High Stochasticity	0.15	0.16	-0.17 0.46	120	0.93	0.352
REM Latency:Fast Volatility	-0.29	0.16	-0.6 0.03	120	-1.8	0.074
N3:Trait Anxiety:High Stochasticity	-0.26	0.23	-0.71 0.18	120	-1.16	0.247
N3:Trait Anxiety:Fast Volatility	-0.33	0.23	-0.78 0.12	120	-1.45	0.149
Trait Anxiety:REM Latency:High Stochasticity	-0.26	0.12	-0.51 -0.02	120	-2.12	**0.036**
Trait Anxiety:REM Latency:Fast Volatility	0.04	0.12	-0.2 0.29	120	0.34	0.738
N3:REM Latency:High Stochasticity	0.41	0.18	0.05 0.77	120	2.23	**0.028**

N3:REM Latency:Fast Volatility	-0.17	0.18	-0.53	0.19	120	-0.92	0.357
**Random effects**	**Variance**				**SD**		
Intercept: participant	0.03				0.17		

Note: REM latency significantly interacted with trait anxiety, particularly with stochasticity. REM latency also interacted with N3 sleep under stochasticity. However, the interactions of REM latency on trait anxiety did not extend to identification of the most rewarding patch, as well as REM latency x N3 interaction was not seen in post-sleep reward learning rates, suggesting that REM latency may not interact at a deeper level on performance i.e., this interaction might be due to chance or needs further systematic manipulation. Thus, we only report N3 x Trait anxiety interaction in the main text as this effect is consistently seen in all the results.

Table S17. Effect of N3 sleep on increase in post-sleep identification of most rewarding patch (Related to STAR Methods).

Linear mixed model (fixed effects: N3 time, REM Latency, Stochasticity, Volatility, Trait Anxiety, Morning State Anxiety ; random effect: participant) results on increase in identification of the most rewarding patch

**Fixed effects**	**β**	**SE**	**95% CI**	***df***	***t***	***p***
Intercept	-0.17	0.14	-0.44 0.11	114.23	-1.22	0.225
N3	-0.02	0.17	-0.35 0.31	124.38	-0.12	0.902
Trait Anxiety	-0.45	0.16	-0.78 -0.13	114.82	-2.74	**0.007**
Dissipation in State Anxiety	0.18	0.18	-0.17 0.54	91.66	1.02	0.310
REM Latency	-0.14	0.15	-0.44 0.15	122.79	-0.96	0.337
High Stochasticity	-0.06	0.14	-0.32 0.21	120.00	-0.42	0.679
Fast Volatility	0.18	0.14	-0.09 0.44	120.00	1.30	0.195
N3:Trait Anxiety	0.44	0.23	-0.01 0.89	106.14	1.92	**0.057**
N3:REM Latency	-0.39	0.18	-0.74 -0.04	117.81	-2.21	**0.029**
N3:Dissipation in State Anxiety	0.17	0.10	-0.03 0.38	40.00	1.72	0.093
N3:High Stochasticity	0.00	0.17	-0.33 0.34	120.00	0.03	0.980
N3:Fast Volatility	0.01	0.17	-0.32 0.34	120.00	0.07	0.945
Trait Anxiety:REM Latency	0.20	0.12	-0.03 0.43	124.78	1.72	0.087
Trait Anxiety:Dissipation in State Anxiety	-0.21	0.11	-0.44 0.02	40.00	-1.86	0.070
Trait Anxiety:High Stochasticity	0.32	0.16	0.01 0.64	120.00	2.00	**0.048**
Trait Anxiety:Fast Volatility	0.13	0.16	-0.19 0.44	120.00	0.78	0.439
Dissipation in State Anxiety:High Stochasticity	-0.04	0.16	-0.34 0.27	120.00	-0.23	0.822
Dissipation in State Anxiety:Fast Volatility	0.08	0.16	-0.23 0.38	120.00	0.49	0.623
REM Latency:High Stochasticity	0.09	0.15	-0.21 0.39	120.00	0.58	0.564
REM Latency:Fast Volatility	-0.17	0.15	-0.47 0.13	120.00	-1.14	0.256
N3:Trait Anxiety:High Stochasticity	-0.10	0.22	-0.53 0.32	120.00	-0.48	0.631
N3:Trait Anxiety:Fast Volatility	-0.49	0.22	-0.92 -0.07	120.00	-2.28	**0.024**
Trait Anxiety:REM Latency:High Stochasticity	-0.15	0.12	-0.38 0.08	120.00	-1.27	0.207
Trait Anxiety:REM Latency:Fast Volatility	-0.05	0.12	-0.28 0.18	120.00	-0.43	0.671
N3:REM Latency:High Stochasticity	0.34	0.17	-0.01 0.68	120.00	1.93	0.056

N3:REM Latency:Fast Volatility	0.02	0.17	-0.33	0.36	120.00	0.09	0.928
**Random effects**	**Variance**				**SD**		
Intercept: participant	0.15				0.38		

Note: REM latency interacted with N3 sleep. For both total rewards and identify most-rewarding patch: greater N3 sleep with lower REM latency helped to increase it post-sleep. However, the interactions of REM latency on trait anxiety did not extend to identification of the most rewarding patch, as well as REM latency x N3 interaction was not seen in post-sleep reward learning rates, suggesting that REM latency may not interact at a deeper level on performance i.e., this interaction might be due to chance or needs further systematic manipulation. Thus, we only report N3 x Trait anxiety interaction in the main text as this effect is consistently seen in all the results.

Table S18. Hierarchical reinforcement learning bayesian analysis model comparison (Related to STAR Methods).

Model Comparisons with model specifications and LOOIC values. Highlighting (in bold) the best model with lowest LOOIC score.

Model	#Parameters	Parameters	LOOIC
banditNarm_delta	2	Learning rate<br>Inverse temperature	23311.32
banditNarm_2par_lapse	3	Reward learning rate<br>Punishment learning rate<br>Lapse	27713.21
banditNarm_4par	4	Reward learning rate<br>Punishment learning rate<br>Reward sensitivity<br>Punishment sensitivity	19850
banditNarm_lapse_decay	6	Reward learning rate<br>Punishment learning rate<br>Reward sensitivity<br>Punishment sensitivity<br>Lapse<br>Decay rate	**19806.39**
banditNarm_singleA_lapse	4	Learning rate<br>Reward sensitivity<br>Punishment sensitivity<br>Lapse	20153.49
banditNarm_lapse	5	Reward learning rate<br>Punishment learning rate<br>Reward sensitivity<br>Punishment sensitivity<br>Lapse	19862.07

Table S19. Winning model prior fits (Related to STAR Methods).
Model prior combinations of the best model. Selecting the best model with the best prior fits which have the lowest LOOIC values without any model convergence/reliability issues. [x]Denotes that the model did not converge, making the LOOIC values for the prior meaningless.

**Priors**	**LOOIC**
	banditNarm_lapse_decay
Session and Block Priors (8)	**19806.39**
Session Priors (2)	20346.54
Block Priors (4)	20580.28
Single Prior (1)	20821.78

Table S20. Effect of N3 sleep on post-sleep reward learning rates (Related to STAR Methods). *Linear mixed model (fixed effects: N3 time, REM Latency, Stochasticity, Volatility, Trait Anxiety, Morning State Anxiety ; random effect: participant) results on increase in post-sleep reward learning rates. Learning rates from individual parameter estimates from the hierarchical Bayesian RL model.*

**Fixed effects**	**β**	**SE**	**95% CI**	***df***	***t***	***p***
Intercept	0.48	0.13	0.23 0.73	107.36	3.82	**0.000**
N3	0	0.15	-0.3 0.29	117.35	-0.03	0.976
Trait Anxiety	0	0.15	-0.3 0.29	107.93	-0.02	0.985
Dissipation in State Anxiety	0.13	0.16	-0.19 0.46	86.14	0.81	0.421
REM Latency	-0.22	0.14	-0.49 0.05	115.76	-1.64	0.104
High Stochasticity	-0.92	0.12	-1.16 -0.69	120	-7.77	**0.000**
Fast Volatility	0.05	0.12	-0.19 0.28	120	0.42	0.677
N3:Trait Anxiety	-0.45	0.21	-0.86 -0.03	99.61	-2.14	**0.035**
N3:REM Latency	0.18	0.16	-0.14 0.49	110.84	1.11	0.270
N3:Dissipation in State Anxiety	-0.04	0.09	-0.23 0.15	40	-0.45	0.658
N3:High Stochasticity	-0.19	0.15	-0.48 0.1	120	-1.29	0.200
N3:Fast Volatility	0.05	0.15	-0.24 0.35	120	0.37	0.712
Trait Anxiety:REM Latency	-0.11	0.1	-0.31 0.1	117.76	-1.04	0.302
Trait Anxiety:Dissipation in State Anxiety	0.01	0.11	-0.21 0.22	40	0.06	0.956
Trait Anxiety:High Stochasticity	-0.07	0.14	-0.35 0.21	120	-0.5	0.621
Trait Anxiety:Fast Volatility	0.07	0.14	-0.21 0.35	120	0.49	0.625
Dissipation in State Anxiety:High Stochasticity	-0.01	0.14	-0.28 0.26	120	-0.11	0.915
Dissipation in State Anxiety:Fast Volatility	-0.04	0.14	-0.31 0.23	120	-0.32	0.749
REM Latency:High Stochasticity	0.01	0.13	-0.25 0.28	120	0.11	0.911
REM Latency:Fast Volatility	0.03	0.13	-0.23 0.29	120	0.24	0.813
N3:Trait Anxiety:High Stochasticity	-0.08	0.19	-0.45 0.29	120	-0.42	0.678
N3:Trait Anxiety:Fast Volatility	0.26	0.19	-0.12 0.63	120	1.36	0.178
Trait Anxiety:REM Latency:High Stochasticity	0.13	0.1	-0.07 0.34	120	1.3	0.198
Trait Anxiety:REM Latency:Fast Volatility	0.06	0.1	-0.15 0.26	120	0.57	0.570
N3:REM Latency:High Stochasticity	-0.11	0.15	-0.42 0.19	120	-0.74	0.46
N3:REM Latency:Fast Volatility	-0.2	0.15	-0.5 0.1	120	-1.3	0.196
**Random effects**	**Variance**			**SD**		

Intercept: participant	0.14	0.38

Table S21. Hierarchical drift-diffusion model comparisons (Related to STAR Methods).
Model Comparisons with model specifications and DIC values. Highlighting (in bold) the best model i.e., with the lowest DIC score. Depends_on column denotes which parameters were fit on which priors. ˣDenotes that the model did not converge (R-hat value greater than 1.05), making the DIC values for the prior meaningless.

**Priors**	**Parameters depends_on**				**DIC**
Session* Block	*v* : session, block	*a* : session, block	*t* : session, block	*z* : session, block	43152.91ˣ
Session*Block	*v* : session, block	*a* : session, block	*t* : session	*z* : session, block	**44878.01**
Session*Block	*v* : session, block	*a* : session, block	*t* : session	*z* : session	44902.26
Session*Block	*v* : session, block	*a* : session	*t* : session	*z* : session	46123.33
Block	*v* : block	*a* : block	*t* : block	*z* : block	47107.52
Block	*v* : block	*a* : block	*t*	*z* : block	48041.03
Block	*v* : block	*a* : block	*t*	*z*	48089.02
Block	*v* : block	*a*	*t*	*z*	48581.39
Session	*v* : session	*a* : session	*t* : session	*z* : session	46700.83
Single	*v*	*a*	*t*	*z*	49100.68

Table S22. Effect of N3 sleep on post-sleep drift-rates (Related to STAR Methods).
Linear mixed model (fixed effects: N3 time, REM Latency, Stochasticity, Volatility, Trait Anxiety, Dissipation in State Anxiety; random effect: participant) results on increase in post-sleep drift-rates.

**Fixed effects**	**β**	**SE**	**95% CI**	***df***	***t***	***p***
Intercept	-0.05	0.14	-0.32 0.22	120.91	-0.34	0.732
N3	0.02	0.16	-0.3 0.34	130.95	0.1	0.922
Trait Anxiety	-0.52	0.16	-0.84 -0.2	121.5	-3.25	**0.002**
Dissipation in State Anxiety	0.06	0.17	-0.29 0.41	97.35	0.34	0.734
REM Latency	-0.13	0.15	-0.42 0.16	129.4	-0.88	0.382
High Stochasticity	-0.24	0.14	-0.51 0.03	120	-1.77	**0.079**
Fast Volatility	0.12	0.14	-0.15 0.39	120	0.89	0.373
N3:Trait Anxiety	0.48	0.22	0.03 0.92	112.64	2.13	**0.035**
N3:REM Latency	-0.38	0.17	-0.72 -0.04	124.49	-2.21	**0.029**
N3:Dissipation in State Anxiety	0.2	0.1	0.01 0.39	40	2.06	**0.046**
N3:High Stochasticity	-0.02	0.17	-0.35 0.31	120	-0.12	0.906
N3:Fast Volatility	-0.04	0.17	-0.38 0.29	120	-0.26	0.798
Trait Anxiety:REM Latency	0.18	0.11	-0.04 0.4	131.34	1.59	0.114
Trait Anxiety:Dissipation in State Anxiety	-0.13	0.11	-0.35 0.08	40	-1.23	0.226
Trait Anxiety:High Stochasticity	0.4	0.16	0.07 0.72	120	2.44	**0.016**
Trait Anxiety:Fast Volatility	0.19	0.16	-0.13 0.51	120	1.15	0.253
Dissipation in State Anxiety:High Stochasticity	-0.03	0.16	-0.34 0.28	120	-0.17	0.869
Dissipation in State Anxiety:Fast Volatility	0.12	0.16	-0.19 0.43	120	0.77	0.444
REM Latency:High Stochasticity	0.09	0.15	-0.22 0.39	120	0.57	0.572
REM Latency:Fast Volatility	-0.21	0.15	-0.51 0.09	120	-1.37	0.173
N3:Trait Anxiety:High Stochasticity	-0.22	0.22	-0.65 0.2	120	-1.03	0.304
N3:Trait Anxiety:Fast Volatility	-0.51	0.22	-0.93 -0.08	120	-2.34	**0.021**
Trait Anxiety:REM Latency:High Stochasticity	-0.11	0.12	-0.35 0.12	120	-0.95	0.347
Trait Anxiety:REM Latency:Fast Volatility	-0.05	0.12	-0.29 0.18	120	-0.46	0.644
N3:REM Latency:High Stochasticity	0.4	0.17	0.05 0.74	120	2.26	**0.025**
N3:REM Latency:Fast Volatility	0.02	0.17	-0.33 0.36	120	0.1	0.922

Random effects	Variance	SD
Intercept: participant	0.11	0.34

Table S23. Average sleep statistics (Related to STAR Methods).
Sleep statistics of participants i.e., mean (standard deviation) of the time spent / percentage in each stage and architecture.

N = 40	Time (min)	% of Total sleep time
Total sleep time (TST)	383.31 (112.18)	
Sleep onset latency (SOL)	31.01 (26.87)	
Wake after sleep onset (WASO)	108.33 (86.42)	
N1	29.09 (20.80)	6.97 (4.28)
N2	196.05 (49.03)	52.82 (10.04)
N3	61.73 (27.56)	16.62 (8.43)
REM	96.45 (47.55)	23.59 (8.65)
REM Latency	120.79 (52.66)	

Note: Beyond N3 sleep, we preregistered hypotheses linking anxiety to other sleep parameters based on prior work. Specifically, we predicted that higher trait anxiety would be associated with fewer REM episodes and longer REM latency, whereas higher state anxiety would be associated with longer sleep onset latency (AsPredicted II, H1c i–ii)[S1]. Firstly, the REM episodes regression model violated assumptions of normality and had to be discarded. Trait anxiety was positively associated with longer REM latency (Linear Regression: β=0.48, 95%CI [0.11, 0.85], t_{34}=2.62, *p*<.05), an effect not observed for state anxiety or subjective sleep quality, indicating a trait-specific contribution to REM latency. However, we did not find state anxiety to affect sleep onset latency (Linear Regression corrected with Wald's test: β=0.05, t_{34}= 0.31, *p*=.8). We also hypothesized that lower morning anxiety would be related to greater power in the delta band during N3 sleep (AsPredicted II H1b)[S2]. As expected, we found a positive effect of dissipation in state anxiety with N3 delta bandpower (Linear Regression: β=0.65, 95%CI [0.15, 1.15], t_{34}= 2.66, *p*<.05).

Supplementary References

1. Horváth, A., Montana, X., Lanquart, J. P., Hubain, P., Szűcs, A., Linkowski, P., and Loas, G. (2016). Effects of state and trait anxiety on sleep structure: A polysomnographic study in 1083 subjects. Psychiatry research, 244, 279–283. https://doi.org/10.1016/j.psychres.2016.03.001
2. Ben Simon, E., Rossi, A., Harvey, A. G., and Walker, M. P. (2020). Overanxious and underslept. Nature human behaviour, 4(1), 100–110. https://doi.org/10.1038/s41562-019-0754-8
